# Comprehensive Analyses of Glucose Metabolism in Glioma Reveal the Glioma-Promoting Effect of GALM

**DOI:** 10.3389/fcell.2021.717182

**Published:** 2022-01-20

**Authors:** Jiacheng Xu, Yuduo Guo, Weihai Ning, Xiang Wang, Shenglun Li, Yujia Chen, Lixin Ma, Yanming Qu, Yongmei Song, Hongwei Zhang

**Affiliations:** ^1^ Department of Neurosurgery, Sanbo Brain Hospital, Capital Medical University, Beijing, China; ^2^ State Key Laboratory of Molecular Oncology, National Cancer Center/National Clinical Research Center for Cancer/Cancer Hospital, Chinese Academy of Medical Sciences and Peking Union Medical College, Beijing, China; ^3^ CAS Key Laboratory of Infection and Immunity, Institute of Biophysics, Chinese Academy of Sciences, Beijing, China

**Keywords:** glioma, glucose metabolism, glucose metabolism-related genes, GALM, EMT

## Abstract

Glioma is the most common tumor with the worst prognosis in the central nervous system. Current studies showed that glucose metabolism could affect the malignant progression of tumors. However, the study on the dysregulation of glucose metabolism in glioma is still limited. Herein, we firstly screened 48 differentially expressed glucose metabolism-related genes (DE-GMGs) by comparing glioblastomas to low-grade gliomas. Then a glucose metabolism-related gene (GMG)-based model (PC, lactate dehydrogenase A (LDHA), glucuronidase beta (GUSB), galactosidase beta 1 (GLB1), galactose mutarotase (GALM), or fructose-bisphosphatase 1 (FBP1)) was constructed by a protein–protein interaction (PPI) network and Lasso regression. Thereinto, the high-risk group encountered a worse prognosis than the low-risk group, and the M2 macrophage was positively relevant to the risk score. Various classical tumor-related functions were enriched by Gene Ontology (GO) and Kyoto Encyclopedia of Genes and Genomes (KEGG) analyses. Since protein GALM was rarely studied in glioma, we detected high expression of GALM by western blot and immunohistochemistry in glioma tissues. And experiments *in vitro* showed that GALM could promote the epithelial-to-mesenchymal transition (EMT) process of glioma cells and could be regulated by TNFAIP3 in glioma cells. Overall, our study revealed the critical role of glucose metabolism in the prognosis of patients with glioma. Furthermore, we demonstrated that GALM was significantly related to the malignancy of glioma and could promote glioma cells’ EMT process.

## 1 Introduction

Glioma is a common intracranial tumor with high mortality and morbidity (Ostrom et al., 2014). For the majority of patients, the traditional treatment is maximum surgical resection with postoperative radiotherapy and chemotherapy, but the average overall survival time is still less than 15 months (Abdul et al., 2018). Therefore, we intended to explore the malignant mechanism of glioma and identify a more feasible prognosis marker.

Recent studies have found that the metabolic reprogramming of tumors could replace the normal metabolic pathway, support the growth and proliferation of cells, and meet the associated bioenergetic and biosynthetic demands (Hanahan and Weinberg, 2011). Moreover, further studies have shown that the genesis and development of the tumor are increasingly dependent on glucose metabolism (Woolf and Scheck, 2015). Studies reported that tumor cells reprogram glucose metabolism and promote tumor growth, proliferation, invasion, and drug resistance through the Warburg effect (Vander Heiden et al., 2009; Liberti and Locasale, 2016; Icard et al., 2018). Hence, the current treatments may be improved by affecting cellular glucose metabolism (Woolf and Scheck, 2015). Many studies have found pathways that affect glucose metabolism in glioma cells; for example, p53 could combine with oncogenes to drive glucose metabolism in glioblastomas (GBMs) (Mai et al., 2017). In GBMs, glucose uptake and cell growth could be promoted by IKKβ and NF-κB signal pathways activated by α-KG (Wang et al., 2019). A study reported that MTORC2 regulates glycolysis of GBMs by increasing c-Myc (Masui et al., 2013). Therefore, understanding the changes in glucose metabolism of gliomas would provide a new strategy for cancer treatment (DeBerardinis and Chandel, 2016).

**FIGURE 1:**
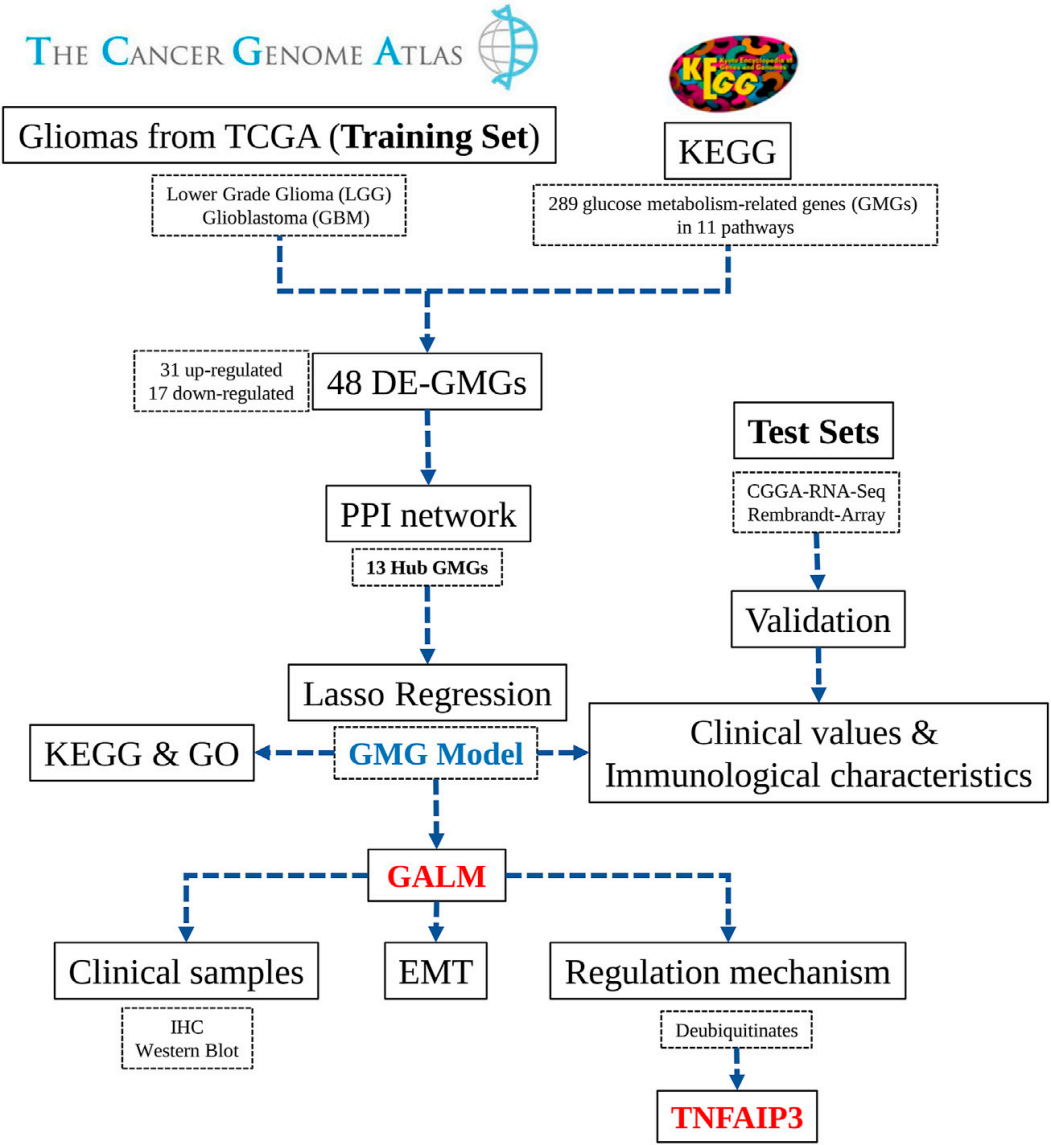
Workflow diagram of this research.

In the study ( Figure 1), we selected 289 glucose metabolism-related genes (GMGs) involved in 11 glucose metabolism-related pathways from the Kyoto Encyclopedia of Genes and Genomes (KEGG) database. Then 48 differentially expressed GMGs (DE-GMGs) were screened between GBMs and low-grade gliomas (LGGs). And the protein–protein interaction (PPI) network was used to identify 13 hub genes. Furthermore, we constructed a GMG model by Lasso regression, including six genes (PC, lactate dehydrogenase A (LDHA), glucuronidase beta (GUSB), galactosidase beta 1 (GLB1), galactose mutarotase (GALM), and fructose-bisphosphatase 1 (FBP1)). Moreover, the underlying biological functions and pathways related to the model were analyzed by functional analyses. Since little is known about GALM in glioma, we focused our study on GALM. We found that GALM was overexpressed in glioma and could promote the epithelial-to-mesenchymal transition (EMT) process of glioma cells. In addition, high expression of GALM could be regulated by TNFAIP3.

## 2 Materials and Methods

### 2.1 Data Acquisition

The TCGA RNAseq (HTSeq-FPKM) data of LGG, GBM, and related clinical information were downloaded from https://portal.gdc.cancer.gov/. The CGGA dataset was acquired from https://www.cgga.org.cn (The REMBRANDT array data was downloaded from GEO database (https://www.ncbi.nlm.nih.gov/geo/query/acc.cgi?acc=GSE108474). Samples with incomplete clinical information were excluded. Finally, the TCGA dataset containing 609 gliomas was set as the training set. The CGGA dataset with 430 gliomas and the REMBRANDT dataset with 278 gliomas were set as test sets.

### 2.2 Patient Tissue Samples

All the clinical specimens were taken from Sanbo Brain Hospital Capital Medical University, and all of them were primary grade II, III, and IV gliomas with reliable pathological diagnoses. The diagnosis, surgery, and postoperative treatment of the patients were followed up. All the tissues were collected from fresh surgical specimens. After surgical separation, we treated them in liquid nitrogen for 30 min and stored them at −80°C for a long time until RNA and protein were extracted. All samples in this study were approved by the Ethics Committee of Sanbo Brain Hospital, Capital Medical University.

### 2.3 Screening of DE-GMGs

Firstly, the RNAseq data of gliomas from TCGA were preprocessed, including background correction, elimination of invalid data, data normalization, and calculation of gene expression. The expression profile of 289 GMGs in 11 pathways was extracted from the matrix. The DE-GMGs were screened by the Limma package via comparing GBMs and LGGs. The cutoff thresholds were intended to be log2|fold change| > 1 and false discovery rate (FDR) < 0.05.

### 2.4 Functional Enrichment Analysis

The GO enrichment analysis of the GMG model was performed on the DAVID dataset (https://david.ncifcrf.gov), including biological process (BP), cellular component (CC), and molecular function (MF), *p* < 0.01, and a count >10 was considered as the cutoff threshold. The KEGG (http://www.genome.ad.jp/kegg/) database was analyzed for possible related pathways of this model, *p* < 0.01, and a count >5 was considered as the cutoff criterion. All results were visualized by R and Cytoscape software (Huang da et al., 2009).

### 2.5 PPI Network and Hub Genes

The PPI network of 48 DE-GMGs was constructed by using the STRING database (http://stringdb.org/) (Szklarczyk et al., 2015). And we removed nodes with an interaction score greater than 0.7 and isolated. Then, we performed two algorithms (MCC and Degree) to screen the hub genes by the CytoHubba plug-in (Chin et al., 2014). All of the above data were visualized using the Cytoscape software (Shannon et al., 2003).

### 2.6 Construction and Validation of the Model

We evaluated the hub genes and determined factors affecting prognosis by univariate Cox regression analysis. And then, we identified the six most valuably predictive genes by lasso regression. Multivariate Cox analysis evaluated the six core GMGs and constructed a risk score prognosis model based on expression value and Cox regression coefficient. Then the median of all risk scores was taken as the cutoff threshold to separate into low- and high-risk groups. The predictive value of this model was evaluated by constructing receiver operating characteristic (ROC) curves. In addition, we also evaluated the predictive value of the model by performing univariate Cox regression analysis for the prognosis of patients.

### 2.7 Cell Culture and Transfection

Ten percent fetal bovine serum (FBS) was added to DMEM (XiGong Biotechnology, China) medium for cell culture under the condition of 37°C and 5% CO_2_. The cells we used included human GBM cells U87, U343, and human embryonic kidney cell HEK-293T. All cell lines were obtained from laboratory-preserved cells. The GALM siRNAs (small-interfering RNA) were purchased from JTS Scientific (Supplementary Table S5). According to the instruction manual, we used Lipofectamine 2000 (Invitrogen, United States) to transfect GALM siRNA into cells. According to the instruction manual, the Neofect DNA transfection reagent (Genomtec, China) was used to transfect the deubiquitinase (DUB) overexpression plasmids. The overexpression plasmids were purchased from Geneppl Technology, Co., Ltd.

### 2.8 Western Blot

We dissolved cells and tissues by using a RIPA buffer supplemented with a protease inhibitor cocktail. Then we separated the protein mixture by SDS-polyacrylamide gel electrophoresis. Therewith, a polyvinylidene fluoride membrane was used to transfer the protein. One hour was enough to seal it with 5% skim milk. And it was necessary to incubate it with the corresponding antibody at 4°C overnight. After that, we washed the membrane with PBST three times and then incubated it with horseradish peroxidase (HRP)-coupled antimouse IgG H&L (W4021, 1:2,000) and antirabbit IgG H&L (W4011, 1: 3,000) for 1 h. Rewash the membrane with PBST three times for 5 min. The binding antibody was detected by a hypersensitive ECL chemiluminescence kit (NCM Biotech), and the image was collected by the chemiluminescence imager Image 800.

The specific primary antibodies used are as follows: GALM (1:500; 16022-1-AP, ProteinTech), E-cadherin (1:500; sc-7870, Santa), Slug (1:500; sc-15391, Santa), Snail (1:500; 3879S, CST), Twist (1:500; ab49254, Abcam), Vimentin (1:500; sc-32322, Santa), and β-Actin (1:5,000; 3700S, CST).

### 2.9 Immunohistochemistry

First, the paraffin slices were dewaxed, hydrated, and incubated at room temperature with an endogenous peroxidase blocker for 20 min. Then antigen repair was performed in a boiled EDTA antigen repair solution (pH 9.0). After that, the pathological section was sealed with sheep serum for 20 min and incubated with GALM (1:50; 16022-1-AP, ProteinTech) antibody at 4°C overnight. The slices were cleaned, the second antibody was incubated, and the color was developed with a DAB chromogenic solution, redyed with hematoxylin, differentiated with 1% hydrochloric acid/75% alcohol, and returned to blue with 1% ammonia. Finally, the slices were dehydrated by gradient alcohol and sealed with neutral resin. The sections were scanned by a gene chip scanner, and five visual fields were randomly selected to determine the immunohistochemical score of GALM positive cells. We recorded the staining intensity as 0 (no staining), 1 (light color), 2 (moderate color), or 3 (deep color). And we recorded the proportion of stained cells as 0 (<5%), 1 (5%–25%), 2 (26%–50%), 3 (51%–75%), or 4(>75%). The formula for calculating the immunohistochemical score was as follows: IHC score = staining intensity × proportion of stained cells. The tissue chips were obtained from Sanbo Brain Hospital Capital Medical University and US Biomax (GL242 B055).

### 2.10 Total RNA Extraction and qRT-PCR

We purchased an RNA Express Total RNA Kit (NCM Biotech) to extract RNA from tissues and cells. And NanoDrop 2000 (Thermo Fisher Scientific, United States) was used to quantify RNA concentration. We used a reverse-transcription kit (Promega) to reverse 2 μg RNA into cDNA. Then the target gene was amplified by 2× qPCR MasterMix (ABM). The relative expression of GALM was calculated using the 2^−ΔΔCt^ method and standardized for actin. The experiments were repeated three times. Sequences of target gene-specific primers are provided in Supplementary Table S6.

### 2.11 Statistical and Survival Analysis

The above data were processed by R software and corresponding packages. We performed the Cox proportional hazard model and the Kaplan–Meier method to evaluate the survival rate. The Glmnet package was used for Lasso–Cox analysis. We used one-way ANOVA and the Student *t*-test to evaluate the differences between each group. Differences with a *p*-value <0.05 were considered statistically significant.

## 3 Results

### 3.1 Screening of DE-GMGs in Glioma

**FIGURE 2:**
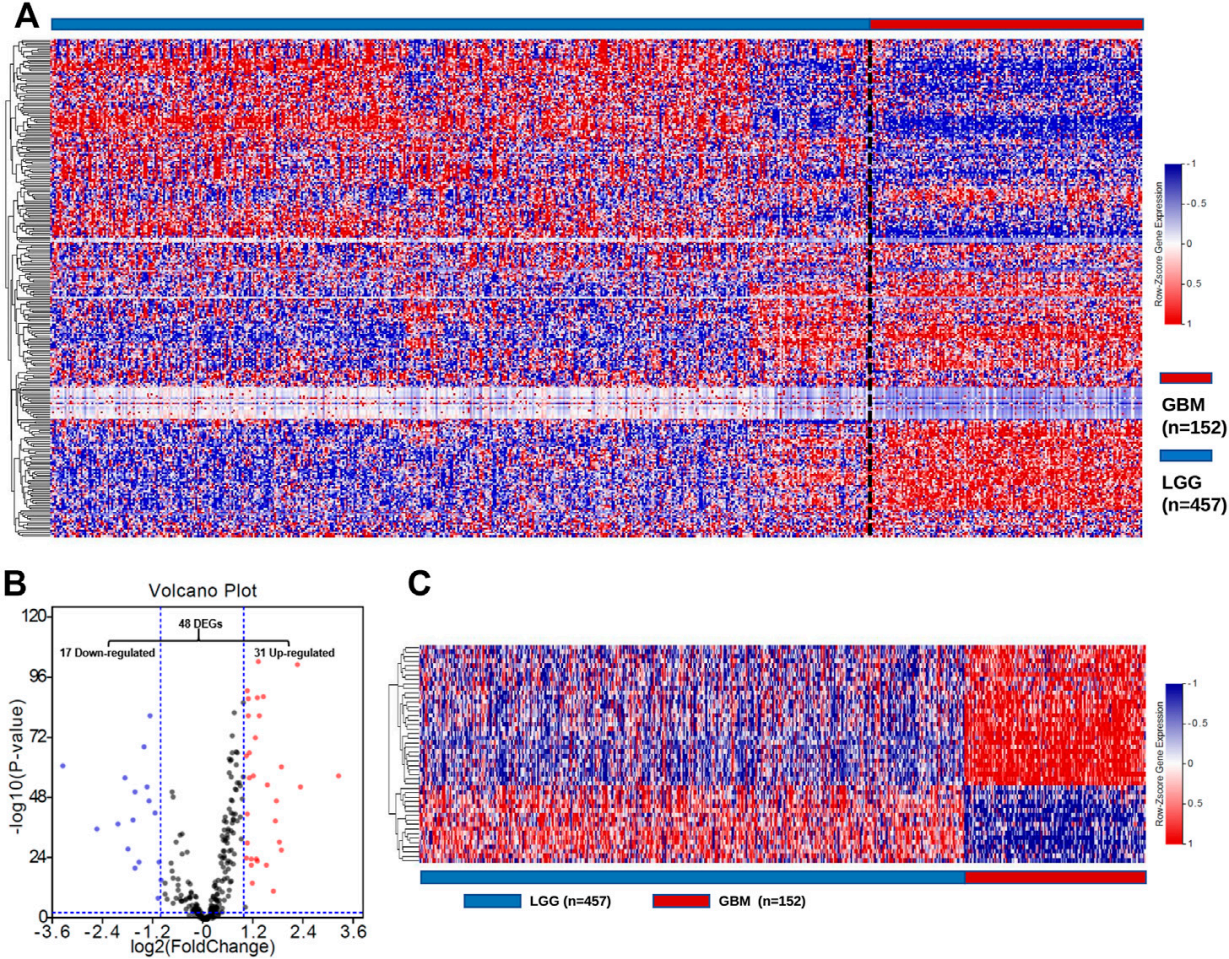
Screening of DE-GMGs. **(A)** Heatmap of 289 GMGs from 11 pathways related to glucose metabolism between GBM and LGG in the TCGA cohort. **(B)** Volcano plots of GMGs by comparing GBM and LGG, log2|fold change| > 1, *p*-value < 0.05. **(C)** Heatmap of 48 DE-GMGs between GBM and LGG.

To characterize the expression of GMGs in gliomas, we selected 11 glucose metabolism-related pathways (Supplementary Table S1) in the KEGG pathway database, including 289 genes (Supplementary Table S2). The expression data of these 289 genes in glioma were obtained from the TCGA database, including 152 GBMs and 457 LGGs. As shown in Figure 2A, GMGs’ expression profile was heterogeneous in gliomas, especially in GBMs. Then we analyzed the expression profile of these 289 genes by comparing GBMs with LGGs. Forty-eight DE-GMGs (Figures 2B,C) were identified by the rank analysis, including 17 downregulated genes and 31 upregulated genes. These results indicated that GMGs were closely related to the malignancy of glioma.

### 3.2 Identification of Hub GMGs

**FIGURE 3:**
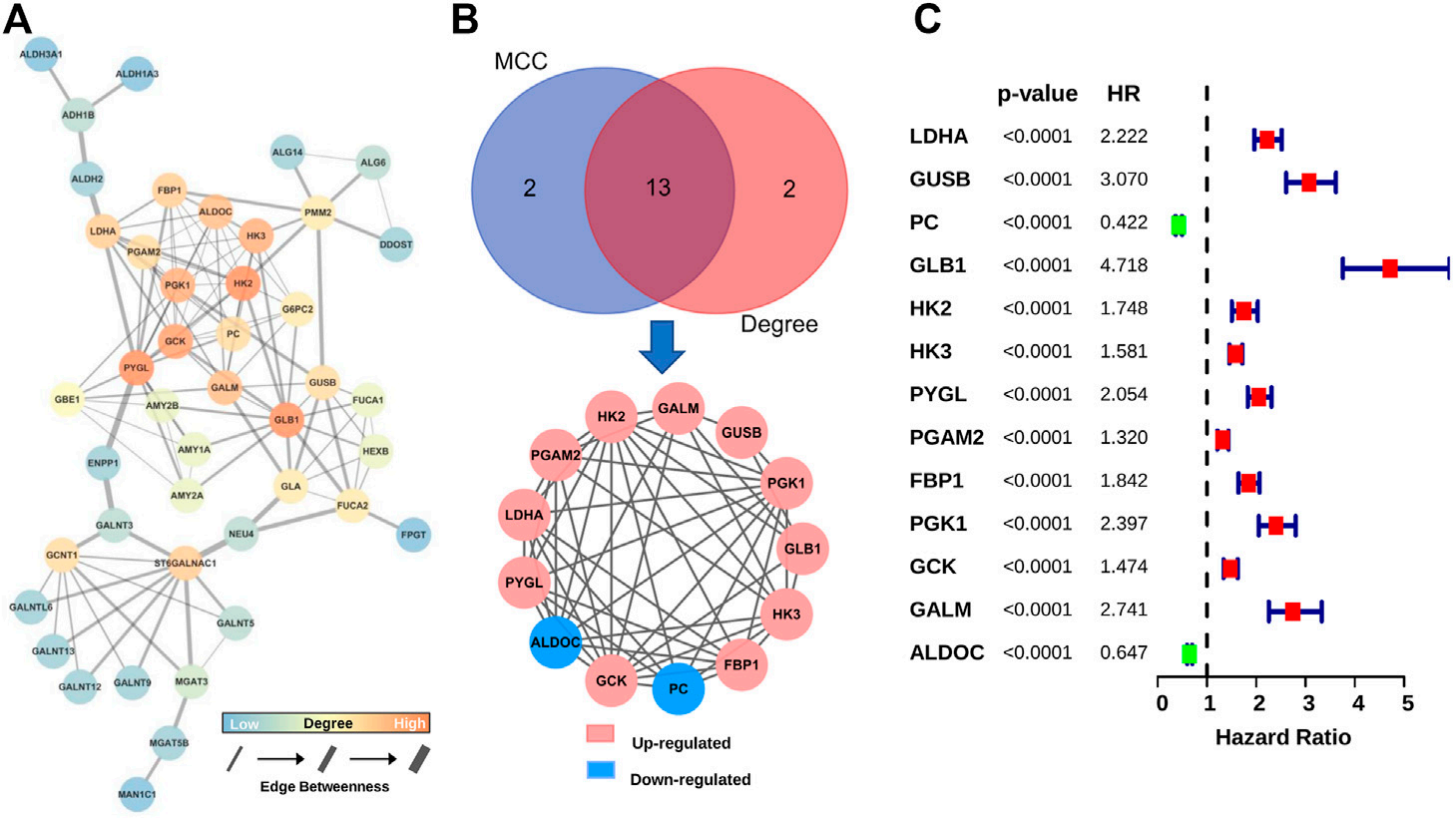
Screening and characteristic analysis of hub genes. **(A)** The PPI network of DE-GMGs. **(B)** Degree and MCC algorithms of CytoHubba were performed to screen the hub genes. **(C)** The prognostic value of each hub gene was visualized with the forest plot of hazard ratios.

In order to obtain GMGs that play the most critical role in glioma malignancy, we constructed a PPI network containing 126 edges and 44 nodes (Figure 3A). Then, 13 hub genes were identified by the MCC and Degree algorithms of CytoHubba, including two downregulated genes (ALDOC and PC) and 11 upregulated genes (HK2, GALM, GUSB, PGK1, GLB1, HK3, FBP1, GCK, PYGL, LDHA, and PGAM2) (Figure 3B). Besides, as shown in the forest map (Figure 3C), all hub genes were significantly associated with gliomas’ prognosis. These results suggested that these 13 hub GMGs played a vital role in glioma development and might be the kernel to judge glioma patients’ prognosis.

### 3.3 Construction of the GMG-Based Model for Glioma

**FIGURE 4:**
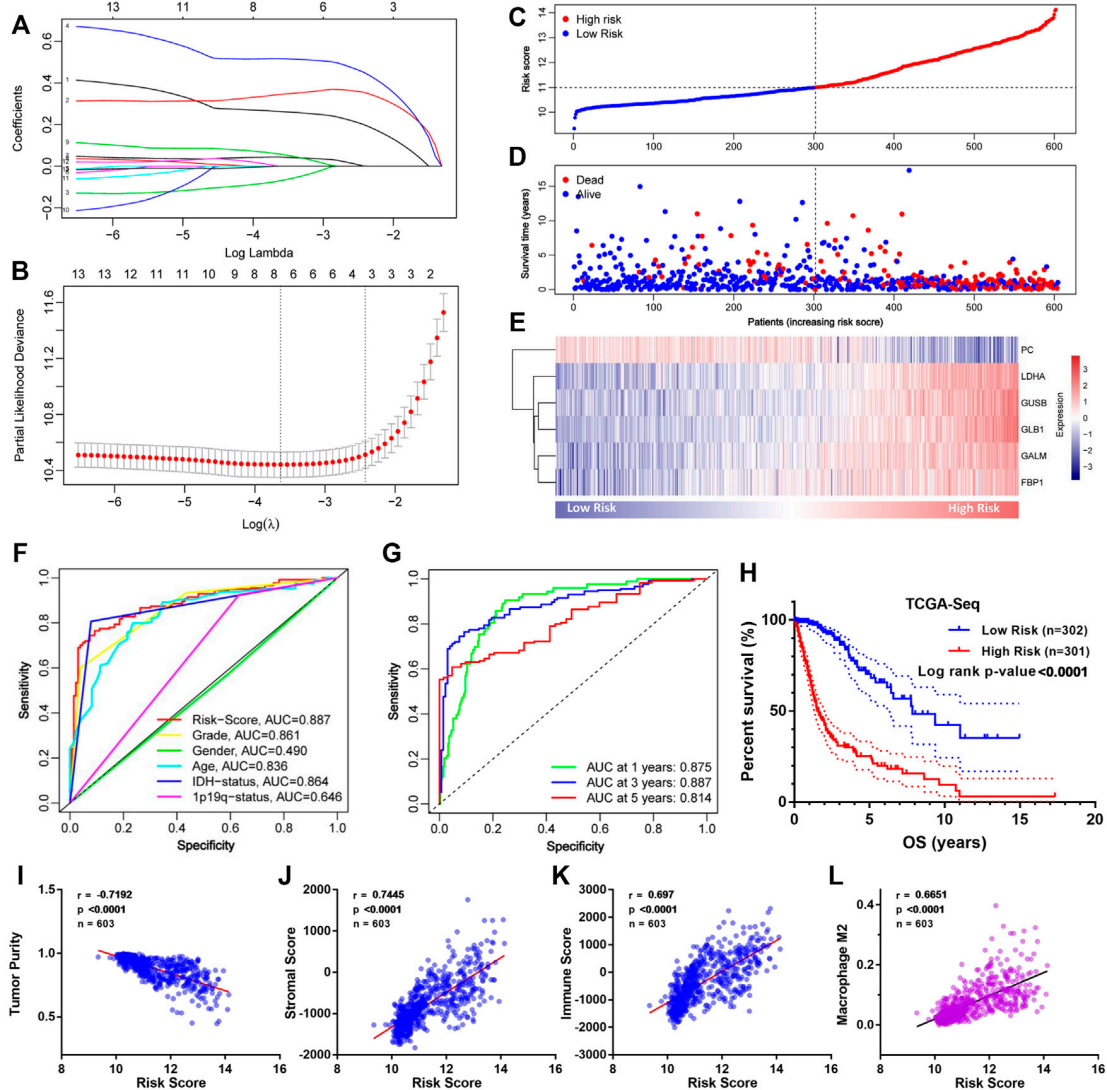
Construction of the GMG-based model for glioma in the TCGA cohort. **(A,B)** Lasso regression and cross-validation of 13 hub genes. **(C)** Grouping of the risk score for each patient. **(D)** Distribution of survival situation of different risk scores for patients. **(E)** Distribution of gene expression panel in the prognostic model. **(F)** Survival-dependent ROC curves of risk score, grade, gender, age, IDH status, and 1p19q status. **(G)** Survival-dependent ROC curves at 1, 3, and 5 years. **(H)** Survival analysis of different risk score groups in gliomas. **(I)** Correlation between risk score and tumor purity. **(J)** Relevance of the risk score to stromal cell content. **(K,L)** Relevance of the risk score to immune cell infiltration analyzed by Pearson test.

To construct an accurate model for analyzing clinical values, six genes (PC, LDHA, GUSB, GLB1, GALM, and FBP1) were identified by lasso regression combined with cross-validation (Figures 4A,B) to optimize the model. Then we constructed the risk score formula (model) according to the regression coefficients of the six genes and the corresponding expression levels. The risk score formula was as follows:

Risk score=0.263×Exp(LDHA)+0.345×Exp(GUSB)−0.072×Exp(PC)+0.517×Exp(GLB1)+0.044×Exp(GALM)+0.059×Exp(FBP1)

As shown in Figure 4C, we divided these patients into a low-risk group (*n* = 302) and a high-risk group (*n* = 301) on the basis of median risk score. The distribution of survival status (Figure 4D) revealed that the high-risk group was confronted with a higher mortality rate than the low-risk group. Likewise, survival analysis (Figure 4H) revealed that the high-risk group encountered a worse prognosis compared to the low-risk group. The expression details of the six GMGs with different risk scores are shown in Figure 4E. In addition, the area under the curve (AUC) value of the ROC curve of the GMG model was 0.887 (Figure 4F), which was higher than that of the other clinicopathological factors, including WHO grades and IDH mutation status. Moreover, as shown in Figure 4G, the AUC values at 1, 3, and 5 years were all greater than 0.8. This evidence showed that the GMG model we constructed had a moderate predictive ability in survival analysis. In addition to tumor cells, there are other factors in tumor tissue, such as immune cells and stromal cells, which affect tumor development. Therefore, we intended to explore the relevance of the risk score to these factors. We found that the risk score was positively relevant to stromal cells and immune cells (Figures 4I–K). Further analysis (Figure 4L) showed that M2 macrophages were positively relevant to the risk score, which in turn plays a cancer-promoting role in a variety of tumors, including gliomas. These results indicated that the GMG model with six GMGs could predict glioma patients’ prognosis and implied an association between glucose metabolism and tumor immunity.

### 3.4 Validation of the GMG Model *via* Independent Datasets

**FIGURE 5:**
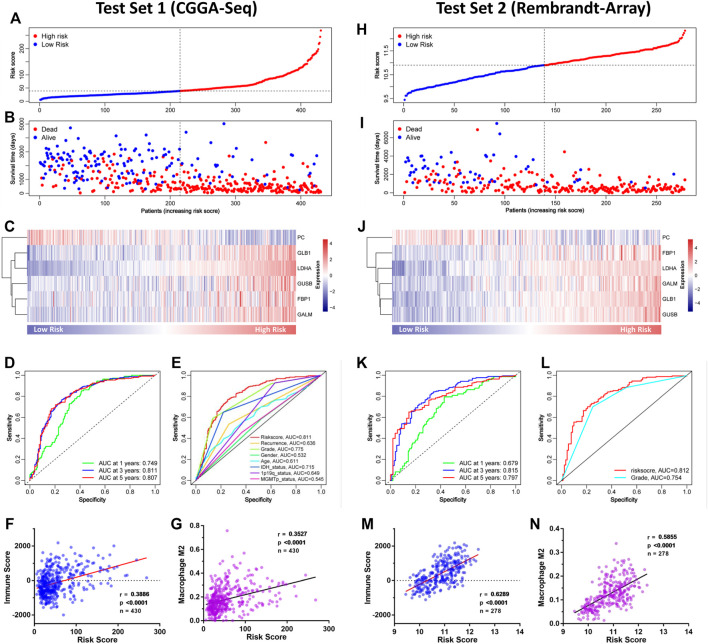
Validation of the GMG model in other independent datasets. **(A–C)** The detailed information of risk score, survival situation, and gene expression profile in the CGGA cohort. **(D)** Survival-dependent ROC curves at 1, 3, and 5 years in the CGGA cohort. **(E)** Survival-dependent ROC curves of risk score, recurrence, grade, gender, age, IDH status, 1p19q status, and MGMTp status in the CGGA cohort. **(F,G)** Relevance of the risk score to immune cell infiltration analyzed by Pearson test in the CGGA cohort. **(H–J)** The detailed information of risk score, survival situation, and gene expression profile in the REMBRANDT array. **(K)** Survival-dependent ROC curves at 1, 3, and 5 years in the REMBRANDT array. **(L)** Survival-dependent ROC curves of grade and risk score in the REMBRANDT array. **(M,N)** Relevance of the risk score to infiltrating immune cells in the REMBRANDT array.

To verify the GMG model’s accuracy and reliability, two independent datasets (CGGA-seq and REMBRANDT array) were acquired as test sets. In the CGGA cohort, the distributions of risk score and gene expression level are shown in Figures 5A,C. And by analyzing different groups, we revealed that the high-risk group encountered higher mortality (Figure 5B) and lower survival (Supplementary Figure S1A) rates compared to the low-risk group. In addition, the AUC of the ROC curve of the model also showed very dependable prediction ability (Figures 5D,E). The CIBERSORT analysis demonstrated that the risk score was positively relevant to immune cells, mainly M2 macrophage (Figures 5F,G). In the REMBRANDT array, similar results were acquired through comprehensive bioinformatics analyses. The distributions of risk score, survival situation, and gene expression profile are shown in Figures 5H–J. And survival analysis (Supplementary Figure S1B) revealed that the high-risk group was also confronted with a worse overall survival (OS) compared to the low-risk group. Moreover, the AUC of the model’s ROC curve also showed an accurate predictive value of the GMG model (Figures 5K,L). As shown in Figures 5M,N, we also found an arresting correlation between risk score and immune cells, especially M2 macrophage. These results suggested that the GMG model was reliable and accurate for gliomas.

### 3.5 Functional Enrichment Analysis

**FIGURE 6:**
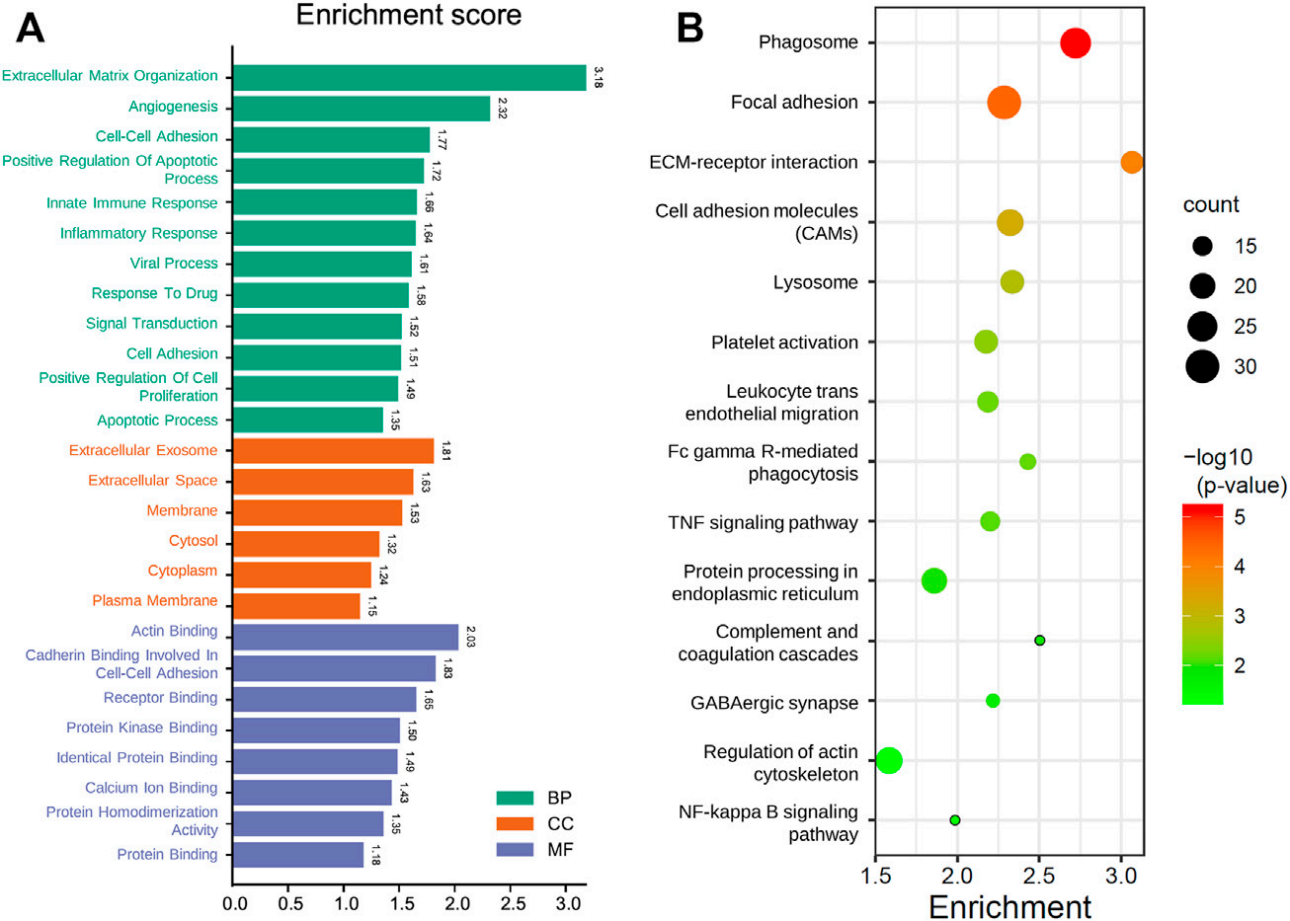
Functional analysis of the GMG model. **(A)** Gene ontology analysis of the identified genes. **(B)** Enriched KEGG pathways of the identified genes (FDR < 0.05 and count > 5).

Since the GMG model played a vital role in predicting patients’ survival with glioma, we explored the underlying biological functions and pathways related to the model. Pearson analysis was applied to acquire 2,875 potential genes related to risk score (*p* < 0.05, *r* > 0.7). Then we conducted GO and KEGG enrichment analyses of these 2,875 genes. As shown in Figure 6A, for the BP, these genes were primarily relevant to the extracellular matrix (ECM) organization, angiogenesis, cell–cell adhesion, and apoptosis. Interestingly, it was also related to innate immune response and inflammatory response. For the CC, these genes were primarily enriched in the cytoplasm, membrane, and extracellular exosome. For the MF, these genes’ functions mainly included receptor binding, protein kinase binding, and protein binding. The KEGG pathway enrichment analysis revealed (Figure 6B) that these genes were mainly connected with the ECM–receptor interaction, phagosome, focal adhesion, cell adhesion molecules (CAMs), leukocyte trans-endothelial migration, and processes associated with cell adhesion and activity. In addition, these genes were also relevant to signaling pathways affecting the tumors’ malignancy, such as the NF-kappa B and TNF signaling pathways. The above results suggested that glucose metabolism might influence the malignant progression of glioma in multiple ways.

### 3.6 Clinical Value of GALM

**FIGURE 7:**
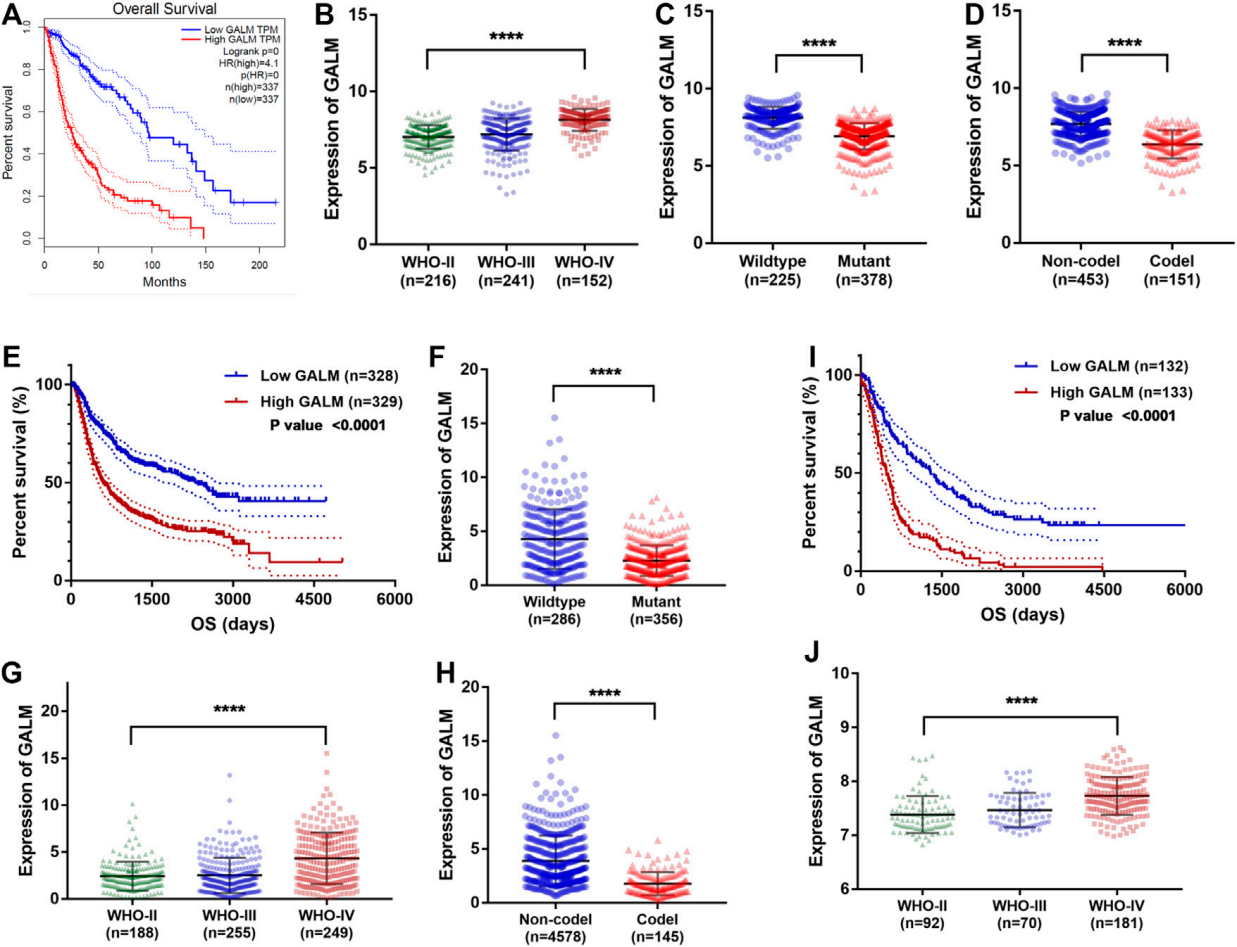
Clinical value of GALM and expression of GALM in different subtypes of glioma. **(A)** In the TCGA cohort, survival analysis of different GALM expression levels in gliomas. **(B–D)** In the TCGA cohort, expression levels of GALM in different glioma subtypes. **(E)** In the CGGA cohort, survival analysis of different GALM expression levels in gliomas. **(F–H)** In the CGGA cohort, expression levels of GALM in different glioma subtypes. **(I)** In the REMBRANDT array, survival analysis of different GALM expression levels in gliomas. **(J)** In the REMBRANDT array, expression levels of GALM in different glioma subtypes.

Through the retrospective investigation of the six GMGs in the model, it was found that little has been known about GALM in glioma. Then we further explored the potential value of GALM. In the TCGA database, survival analysis revealed that the patients with high expression of GALM encountered worse prognosis compared to patients with low expression of GALM (Figure 7A). Also, the expression of GALM showed significant differences in different WHO grades (Figure 7B). And compared to that in IDH mutant gliomas, we found that GALM’s expression in IDH wild-type gliomas was overexpressed (Figure 7C). A similar high expression of GALM was also found in 1p/19q non-codeletion gliomas (Figure 7D). Furthermore, we also validated these results in the CGGA dataset (Figures 7E–H) and REMBRANDT array (Figures 7I,J). These results indicated that GALM’s expression was significantly relevant to gliomas’ prognosis and malignancy, implying that GALM might regulate the malignant progression of gliomas.

### 3.7 Validation of GALM in Clinical Samples

**FIGURE 8:**
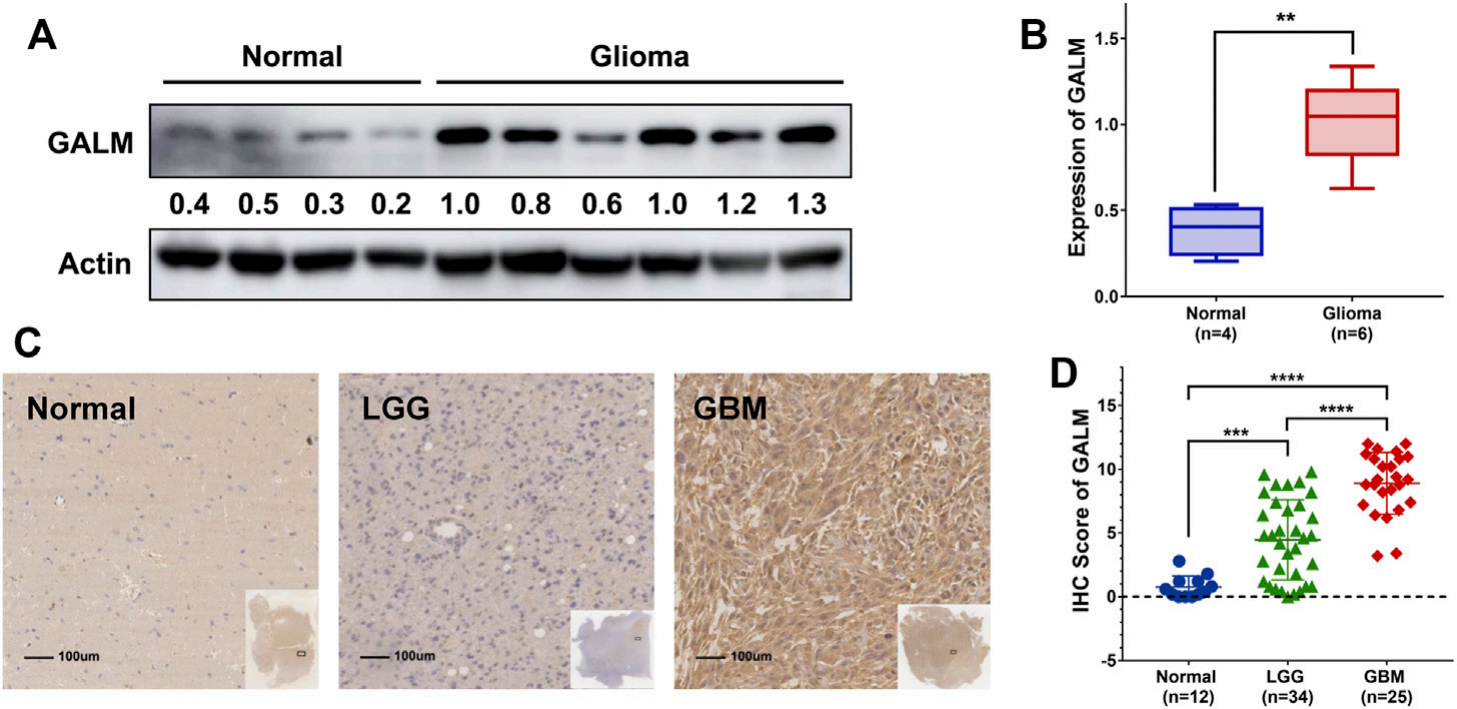
The expression of GALM in clinical samples. **(A,B)** In normal brain and glioma tissues, the expression of GALM was detected by Western blot. **(C,D)** IHC staining of GALM in human normal brain tissues, LGGs, and GBMs; ** represents *p* < 0.01, *** represents *p* < 0.001, and **** represents *p* < 0.0001.

Due to the remarkable clinical significance of GALM in glioma, we intended to further confirm the expression of GALM by Western blot and qRT-PCR in clinical specimens. As shown in Figures 8A,B (Supplementary Figure S2A), a higher expression level of GALM was observed in gliomas compared to normal brain tissues. Furthermore, a larger sample was examined by IHC. As shown in Figures 8C,D (Supplementary Figures S2B–D), GALM was significantly overexpressed in high-grade and IDH wild-type gliomas, which supported data analysis results. These results proved that GALM was overexpressed in gliomas and was closely related to the malignancy of gliomas.

### 3.8 GALM Could Promote the EMT Process of Glioma Cells

**FIGURE 9:**
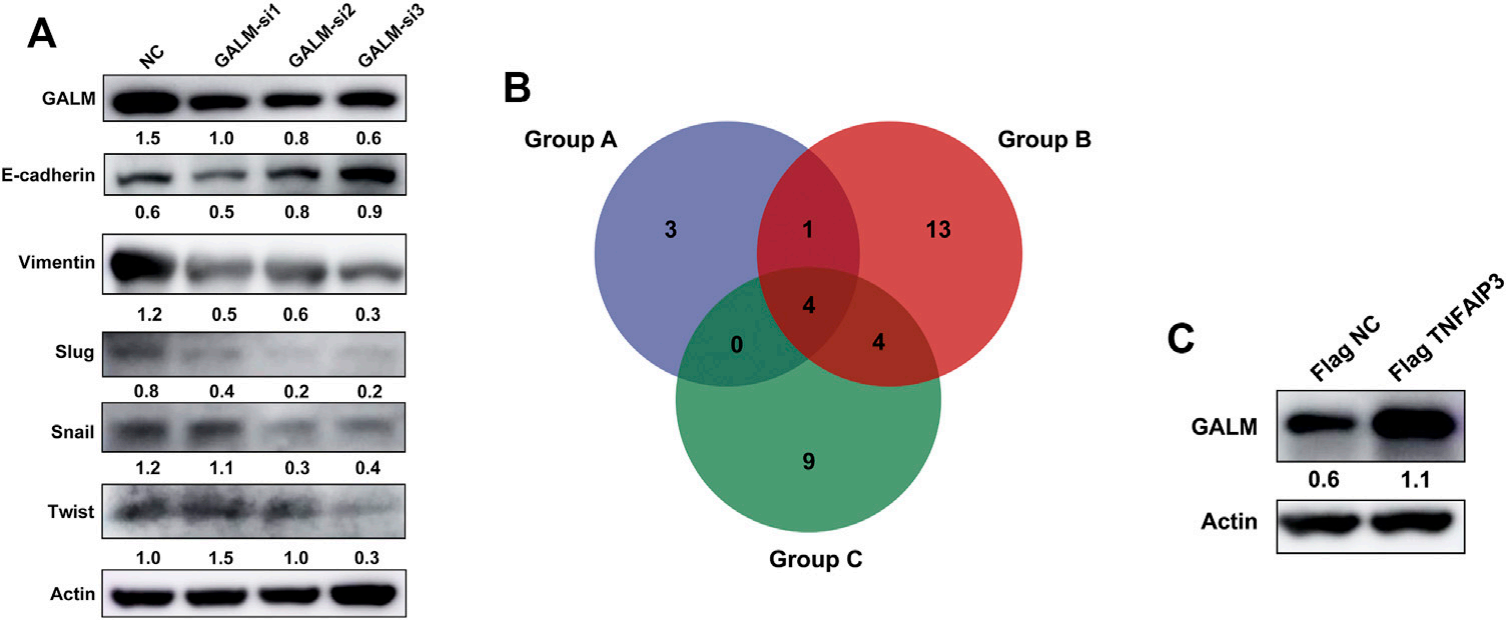
Function and regulatory mechanisms of GALM in glioma cells. **(A)** The expression level of GALM, actin, and EMT biomarkers (E-cadherin, Slug, Snail, Twist, and Vimentin) in U87 cells transfected with siNC or three different siRNAs. **(B)** Venn diagram of Groups A, B, and C (Group A for overexpressed DUBs in glioma, Group B for DUBs positively correlated with GALM in glioma, and Group C for DUBs screened by western blot, which could upregulate the expression of GALM). **(C)** The expression of GALM and actin in U343 cells transfected with Flag NC and Flag TNFAIP3.

In the functional enrichment analysis, plenty of genes were enriched in processes and pathways related to cell adhesion, which has been recognized to be regulated by EMT (Bergeman et al., 2016; O’Connor et al., 2016; Reher et al., 2017; Porretti et al., 2018; Shi et al., 2020). After knocking down GALM with siRNA, we observed that the EMT process of glioma cells was significantly inhibited (Figure 9A; Supplementary Figure S3A). Then, we explored the mechanism for the overexpression of GALM in glioma. The regulation of protein deubiquitination was considered first. By analyzing the expression levels of DUBs (Supplementary Figure S3C) and their correlation with GALM in gliomas (Supplementary Figures S4; Supplementary Table S3), combined with *in vitro* experiments (Supplementary Figure S3B), four DUBs were selected, namely, USP18, TNFAIP3, USP39, and USP38 (Figure 9B; Supplementary Table S4). Among them, TNFAIP3 correlated most significantly with GALM with *R* = 0.52. Notably, the expression of GALM increased significantly after overexpression of TNFAIP3 in the glioma cell (Figure 9C).

In conclusion, GALM was overexpressed in glioma and could promote the EMT process of glioma cells. In addition, high expression of GALM could be regulated by TNFAIP3.

## 4 Discussion

Glioma is the most common malignant intracranial tumor with little long-term treatment effect (Seyfried et al., 2015; Cai et al., 2018). Although the treatments have been improved (Omuro and DeAngelis, 2013), the existing treatments have a limited effect on the progression of the disease and the survival of patients (Poff et al., 2019). Therefore, in order to explore effective treatment strategies, it is essential to study the malignant mechanisms of gliomas. Metabonomics is a promising field of precision medicine and drug discovery. With the rapid development of metabonomics, metabolic changes could reveal effective new molecular intervention targets (Pandey et al., 2017). Relatively speaking, in the field of neuro-oncology, although significant progress has been made in metabonomics, the study on how brain tumors reprogram metabolic pathways is still limited (Venneti and Thompson, 2017). Among them, the reprogramming of glucose metabolism is a promising strategy for the treatment of gliomas (Lu et al., 2020). Therefore, we intended to study the potential changes in glucose metabolism of gliomas and to identify a reliable prognosis marker. In this study, we comprehensively analyzed the 289 genes contained in 11 glucose metabolism-related pathways in the TCGA database, constructed a GMG model based on DE-GMGs, and proved the clinical values and immunological characteristics of the model. Furthermore, we proved that the expression of GALM was overexpressed in gliomas and that GALM could promote the EMT process of glioma cells. In addition, the expression of GALM could be regulated by TNFAIP3.

First of all, the comprehensive analyses revealed that there was a noteworthy heterogeneity in the expression profile of GMGs between GBMs and LGGs. And plenty of GMGs were related to the prognosis. These results suggested that GMGs changed and perhaps played an important role in high-grade gliomas. The studies of Xu et al. (2017) and Xiao et al. (2018) also reported that GMGs, such as GLUT1 and HK2, have changed expression and performed important functions in tumors.

In order to clarify the role of DE-GMGs in gliomas, we constructed a GMG model. A risk score formula (model) was constructed according to the regression coefficient and the corresponding expression level of genes. Then we analyzed and verified the model’s clinical value and immunological characteristics in multiple datasets. Data analysis revealed that a high-risk score possessed a worse prognosis. And immune cells, especially the M2 macrophage, was positively correlated with the risk score. In the tumor microenvironment (TME), the most crucial component is tumor-associated macrophages (TAMs) (Choi et al., 2018). And the TAMs could acquire polarized M2 phenotype driven by various cytokines (Mantovani et al., 2002). Studies have reported that these polarized cells play a key role in tumors (Yang et al., 2020). The study of Chen et al. (2017) showed that the M2 macrophage promotes the metastasis of gastric cancer and breast cancer through the secretion of the CHI3L1 protein. The study of Yang et al. (2018) reported that M2 macrophage polarization is promoted through the Wnt/β-catenin signal pathway between tumor cells and macrophages, which in turn promotes the malignant progression of tumors. Combined with these studies, our results showed that these DE-GMGs significantly affected the prognosis of glioma patients, possibly by regulating the immune mechanism.

Subsequently, the functional analysis of the GMG model revealed how it might play a role, including the “NF-kappa B signaling pathway,” “cell–cell adhesion,” “TNF signaling pathway,” and “leukocyte transendothelial migration.” Our analysis also reported that the innate immune response, the inflammatory response, might be involved in the genesis and development of glioma, revealing the potential immunomodulatory mechanism of GMGs.

In addition, this model contained six GMGs, namely, PC, LDHA, GUSB, GLB1, GALM, and FBP1. Pyruvate carboxylase (PC) exists in the mitochondria and is a member of the biotin-dependent carboxylase family (Wallace et al., 1998). Pyruvate is carboxylated to oxaloacetic acid *via* ATP-dependent pyruvate carboxylase (PC) to supplement the tricarboxylic acid cycle (Cheng et al., 2011). In addition, oxaloacetic acid could also be used to synthesize other compounds, including glucose, fats, some amino acids and their derivatives, and some neurotransmitters (Wallace et al., 1998). Recent studies showed that PC is abnormally expressed and plays an essential role in many tumors, such as ovarian cancer (Shang et al., 2020), lung cancer (Sellers et al., 2015), gallbladder cancer (Ma et al., 2016), and breast cancer (Shinde et al., 2018), and it was proved that PC could participate in tumor proliferation, metastasis, and invasion (Sellers et al., 2015; Christen et al., 2016; Shinde et al., 2018; Lao-On et al., 2020). Moreover, a small molecular inhibitor, ZY-444, was reported to target PC to inhibit the proliferation of breast cancer (Lin et al., 2020). A study showed that PC is overexpressed in non-small-cell lung cancer and could promote tumor proliferation (Sellers et al., 2015). On the contrary, it was interesting that our analysis proved that the expression of PC in glioma was downregulated, which indicated that PC might play different functions through different mechanisms in various tumors. LDHA belongs to the lactate dehydrogenase family, participates in the vital process of glycolysis, and promotes glycolysis by catalyzing the conversion of pyruvate to lactic acid (Cai et al., 2019). A recent study showed that LDHA weakens the immune monitoring of T cells and NK cells to tumors by promoting lactic acid production (Brand et al., 2016). MiR-30a-5p could inhibit growth and metastasis by inhibiting the LDHA-mediated Warburg effect in breast cancer (Li et al., 2017). Phosphorylation-mediated LDHA activation promoted cancer cell invasion and metastasis (Jin et al., 2017). Consistent with our analysis results, a study showed that the expression of LDHA is relevant to the malignancy of tumors and could affect the proliferation, apoptosis, and chemical sensitivity of temozolomide in glioma cells (Di et al., 2018). However, no suitable LDHA inhibitor has been found for tumor therapy (Valvona et al., 2016). GUSB is an essential lysosomal enzyme, which participates in the degradation of glycosaminoglycans. And GUSB deficiency could cause mucopolysaccharidosis VII (MPS VII) (Vogler et al., 2003; Bigg et al., 2013). The studies of Xie, et al. (2014a) and Xie, et al. (2014b) reported that GUSB is abnormally expressed in colorectal cancer and is relevant to abnormal methylation. GLB1 is a lysosomal exoglycosidase involved in the catabolism of glycoconjugates and could affect the senescence of cancer cells (Vidya et al., 2020). Lack of GLB1 caused lysosomal storage disorder and led to G(M1) gangliosidosis (Caciotti et al., 2005). GALM is a mutarotase involved in the mutual transformation of beta-d-galactose and alpha-d-galactose in galactose metabolism (Timson and Reece, 2003). GALM participates in the first step of the Leloir pathway and eventually metabolizes beta-d-galactose to glucose 1-phosphate in the liver (Wada et al., 2019). A study showed that GALM also has an effect on d-glucose, but its effect is not as evident as that on galactose (Timson and Reece, 2003). All-*trans*-retinoic acid (RA) is a vital regulator of GALM in myeloid-monocytic cells (Pai et al., 2007). FBP1 was reported as a metabolic tumor suppressor factor in hepatocellular carcinoma Cancer Discovery (2020), and the deletion of FBP1 could promote tumor growth by affecting crosstalk between hepatocyte metabolism and HSC senescence (Li F. et al., 2020). Similarly, FBP1 was proven to inhibit tumor progression in cholangiocarcinoma (CCA) (Zhao et al., 2018), prostate cancer (PCA) (Zhang et al., 2019), and lung adenocarcinoma (LUAD) (Li L. et al., 2020). However, it was noteworthy that a study in gliomas reported that the expression of FBP1 is positively relevant to the c-Myc level and tumor proliferation (Ding et al., 2015). It was consistent with our results, indicating that FBP1 might play different functions in gliomas. In addition, FBP1 could also affect the function of immune cells. For example, for NK cells, overexpression of FBP1 could lead to dysfunction by inhibiting glycolysis (Cong et al., 2018).

On account of the lack of research on protein GALM in gliomas, we intended to explore the function of GALM in glioma further. Bioinformatic analyses revealed that the poor prognosis happened in glioma patients with high expression of GALM and that the expression of GALM was also related to the malignancy of gliomas. Then we further verified the expression of GALM by western blot, qRT-PCR, and IHC in glioma samples. The above evidence suggested that GALM could be used to estimate the prognosis in glioma. Furthermore, we demonstrated that knocking down the expression of GALM could affect the EMT process of glioma. Moreover, TNFAIP3 could regulate the expression level of GALM. Therefore, we proposed that highly expressed GALM maintained by TNFAIP3 could promote the malignancy of glioma by regulating the EMT process.

However, there are still some limitations in our study. Our study lacked the verification of *in vivo* experiments. And we were required to expand the amount of data because of the limited specimen capacity. The research on the related mechanism was not enough.

In summary, our study revealed the significance of glucose metabolism in gliomas and provided a model composed of six GMGs. Furthermore, we demonstrated that GALM could promote the EMT process of glioma cells and was significantly related to the malignant degree of glioma. Moreover, TNFAIP3 could regulate the high expression of GALM. Our study might provide potential targets for the diagnosis and treatment of glioma.

## Data Availability

The original contributions presented in the study are included in the article/Supplementary Material, further inquiries can be directed to the corresponding authors.

## Abbreviations

AUC, area under curve; BP, biological process; CC, cellular component; DE-GMGs, differentially expressed glucose metabolism-related genes; EMT, epithelial-to-mesenchymal transition; GBM, glioblastoma; GMGs, glucose metabolism-related genes; GO, gene ontology; IHC, immunohistochemistry; KEGG, Kyoto Encyclopedia of Genes and Genomes; MF, molecular function; ROC, receiver operating characteristic; TAM, tumor-associated macrophage; TME, tumor microenvironment.
