# Supplementary material for: Comprehensive Analyses of Glucose Metabolism in Glioma Reveal the Glioma-Promoting Effect of GALM
Source: Front Cell Dev Biol. 2022 Jan 20;9:717182. doi: 10.3389/fcell.2021.717182 (PMC8811465; doi:10.3389/fcell.2021.717182)
Supplement: Supplementary file 1 [file DataSheet1.ZIP › Supplementary Information/Supplementary Information.docx]

Supplementary Material


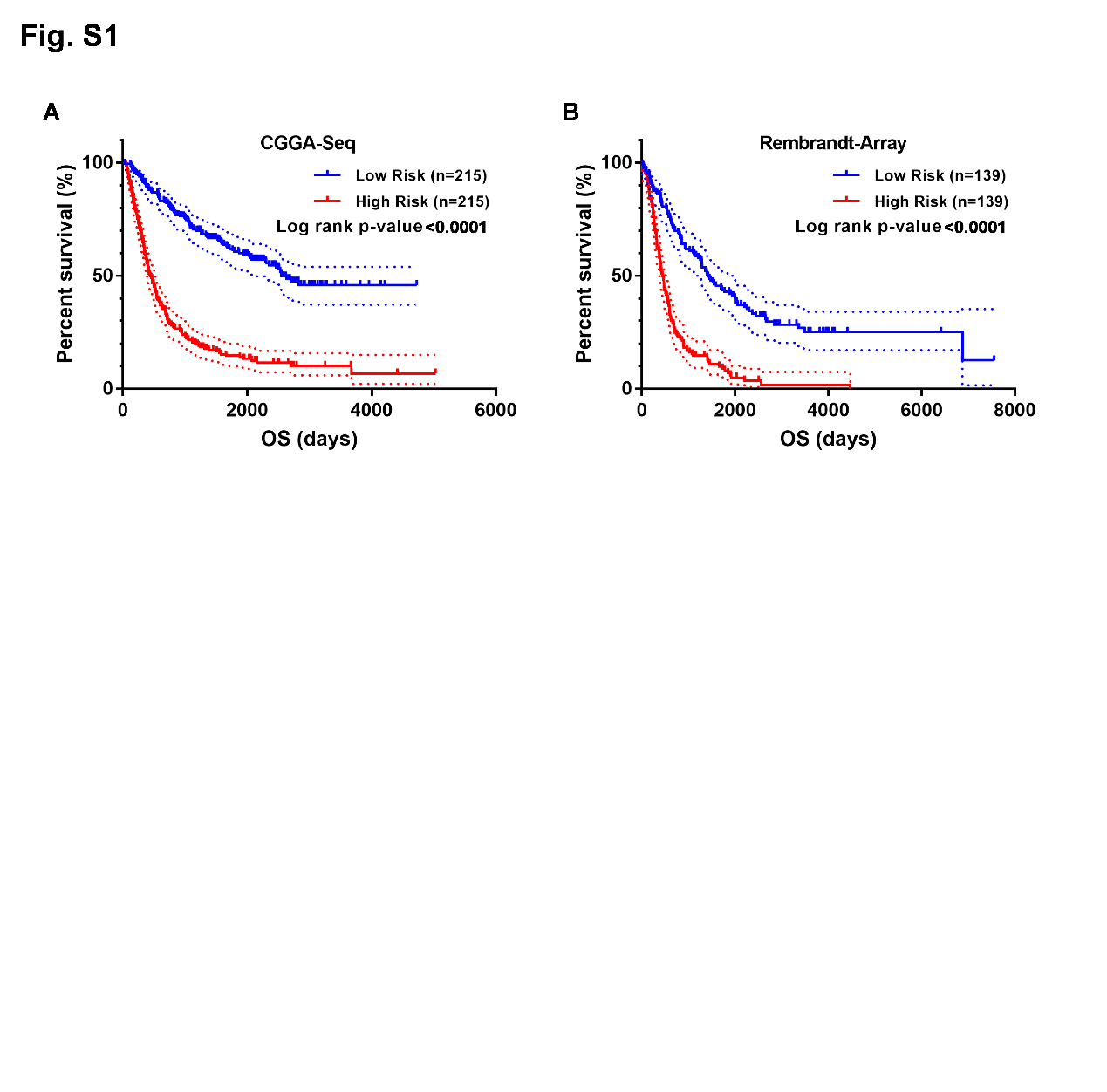


**Figure S1, related to Figure 5.**

**(A)** Survival analysis of gliomas with different risk score groups in the CGGA cohort. **(B)** Survival analysis of gliomas with different risk score groups in the Rembrandt-Array.


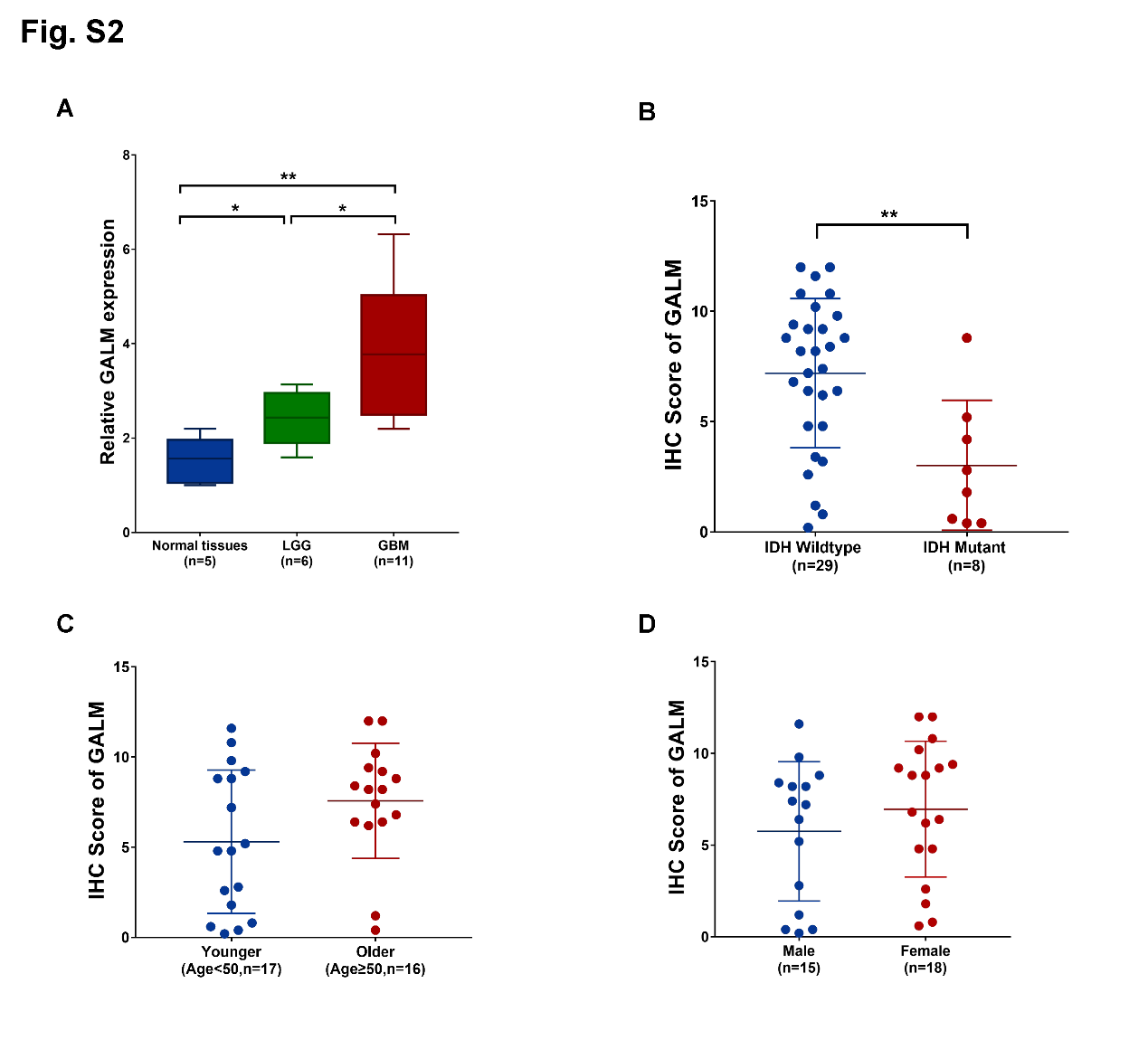


**Figure S2, related to Figure 8. (A)** qRT-PCR to determine the expression of GALM in normal brain tissues, low-grade gliomas and glioblastomas; *P < 0.05; **P < 0.01.**(B)** IHC score of GALM of IDH wildtype and IDH mutant gliomas. **(C)** IHC score of GALM in different ages. **(D)** IHC score of GALM in different genders.


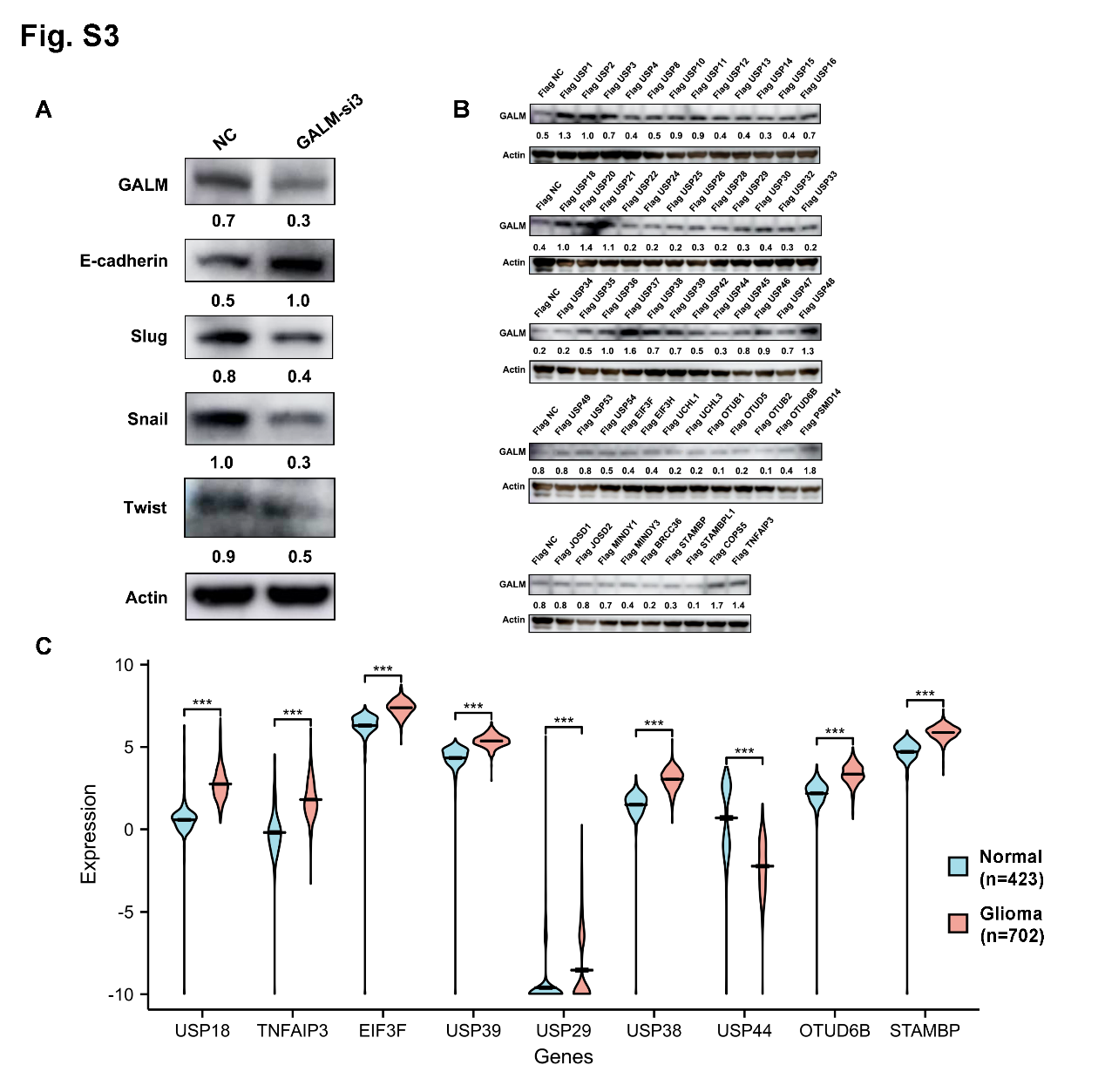


**Figure S3, related to Figure 9.**

**(A)** The expression level of GALM, Actin, and EMT biomarkers (E-cadherin, Slug, Snail, Twist) in U343 cells transfected with siNC and GALM-si3. **(B)** The expression level of GALM in HEK-293T cell transfected with plasmid overexpressing deubiquitinases. **(C)** Differential Expression of deubiquitinases (USP18, TNFAIP3, EIF3F, USP39, USP29, USP38, USP44, OTUD6B, STAMBP) in gliomas and normal brain tissues from TCGA and GTEx datasets; ***P < 0.001.


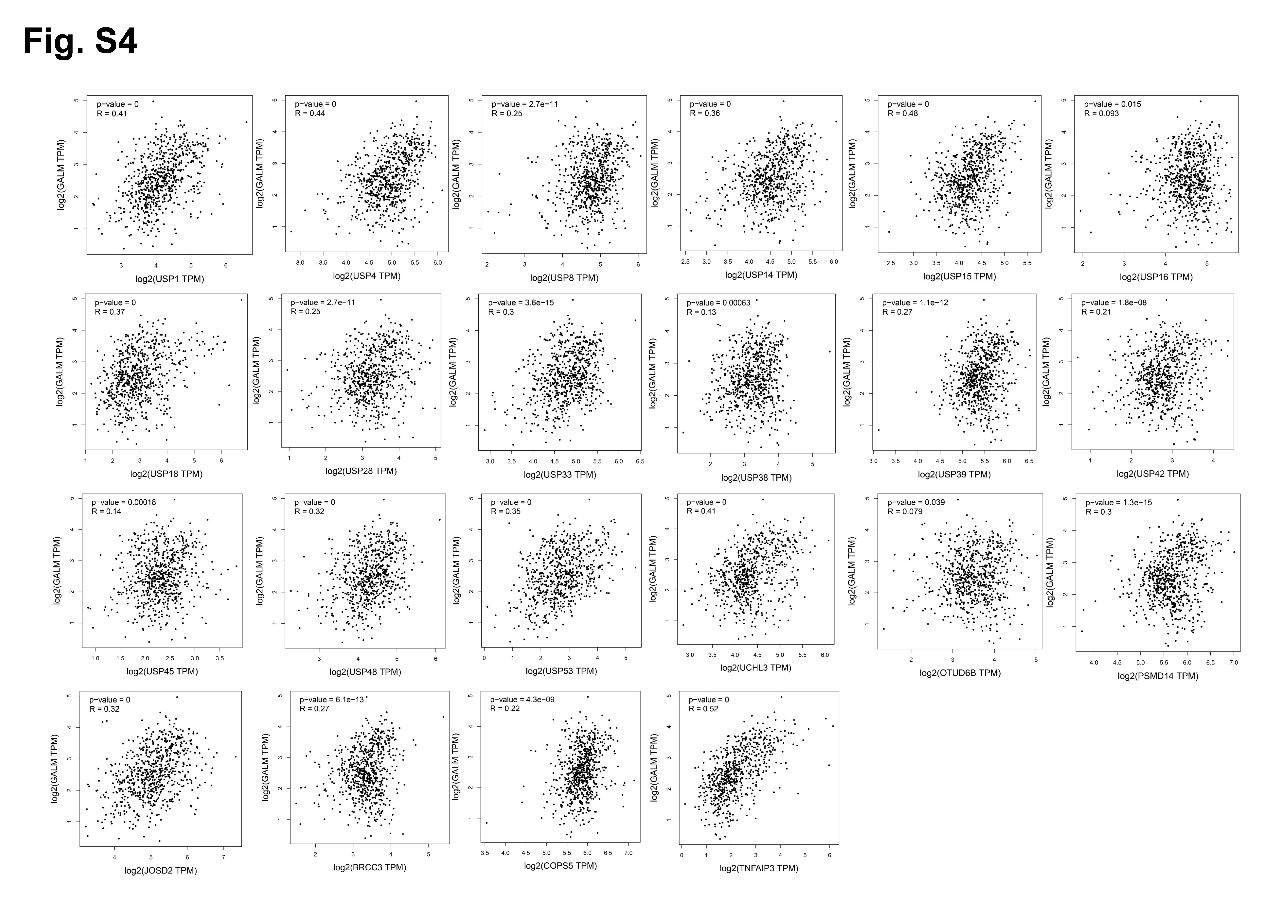


**Figure S4, related to Figure 9.** Correlation analysis showed that the deubiquitinases (USP1, USP4, USP8, USP14, USP15, USP16, USP18, USP28, USP33, USP38, USP39, USP42, USP45, USP48, USP53, UCHL3, OTUD6B, PSMD14, JOSD2, BRCC36, COPS5, TNFAIP3) positively correlated with GALM in the gliomas from TCGA.


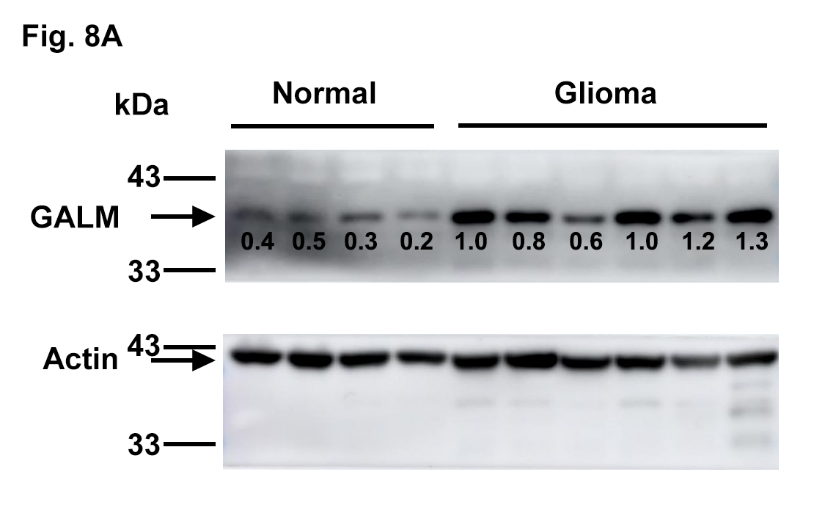


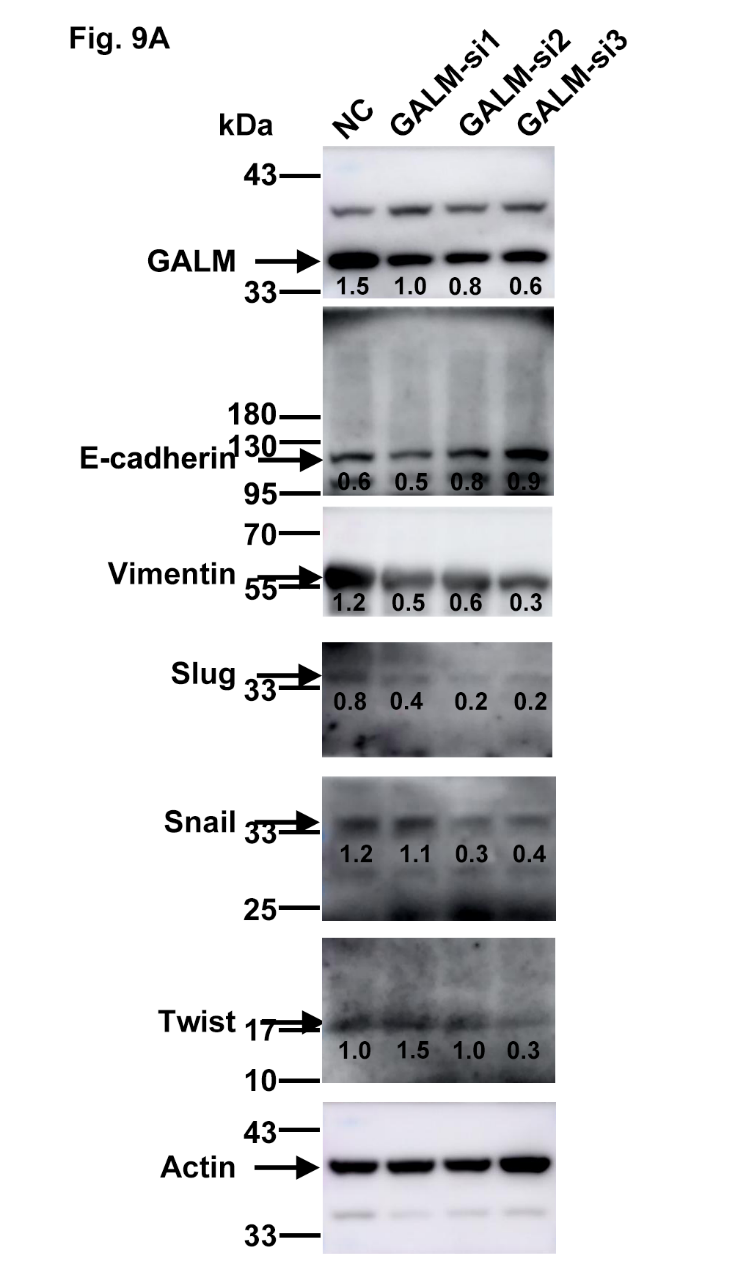

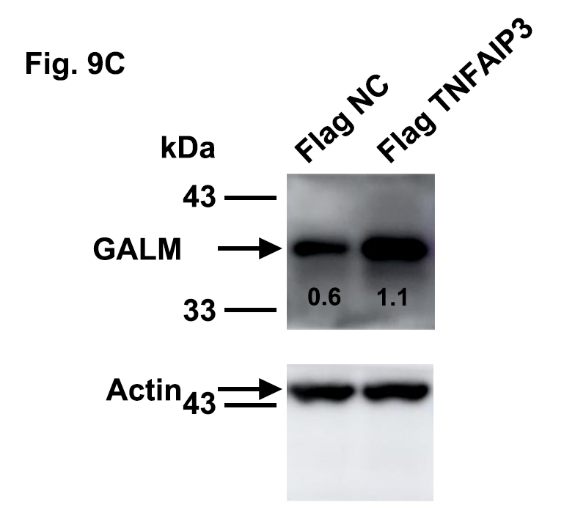


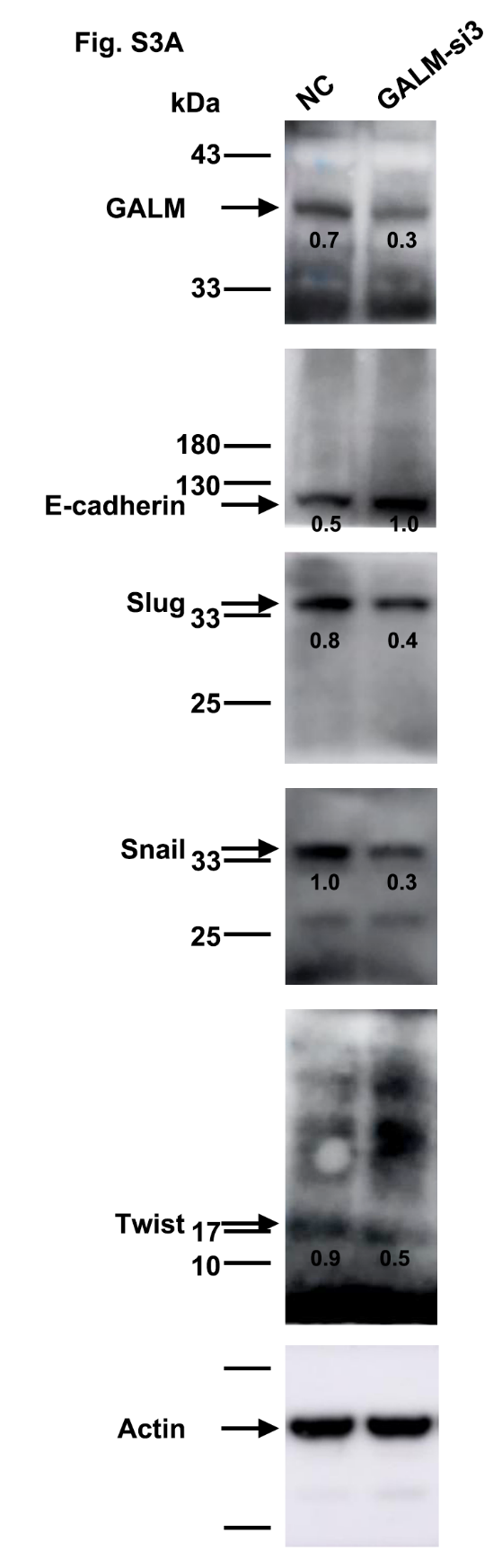


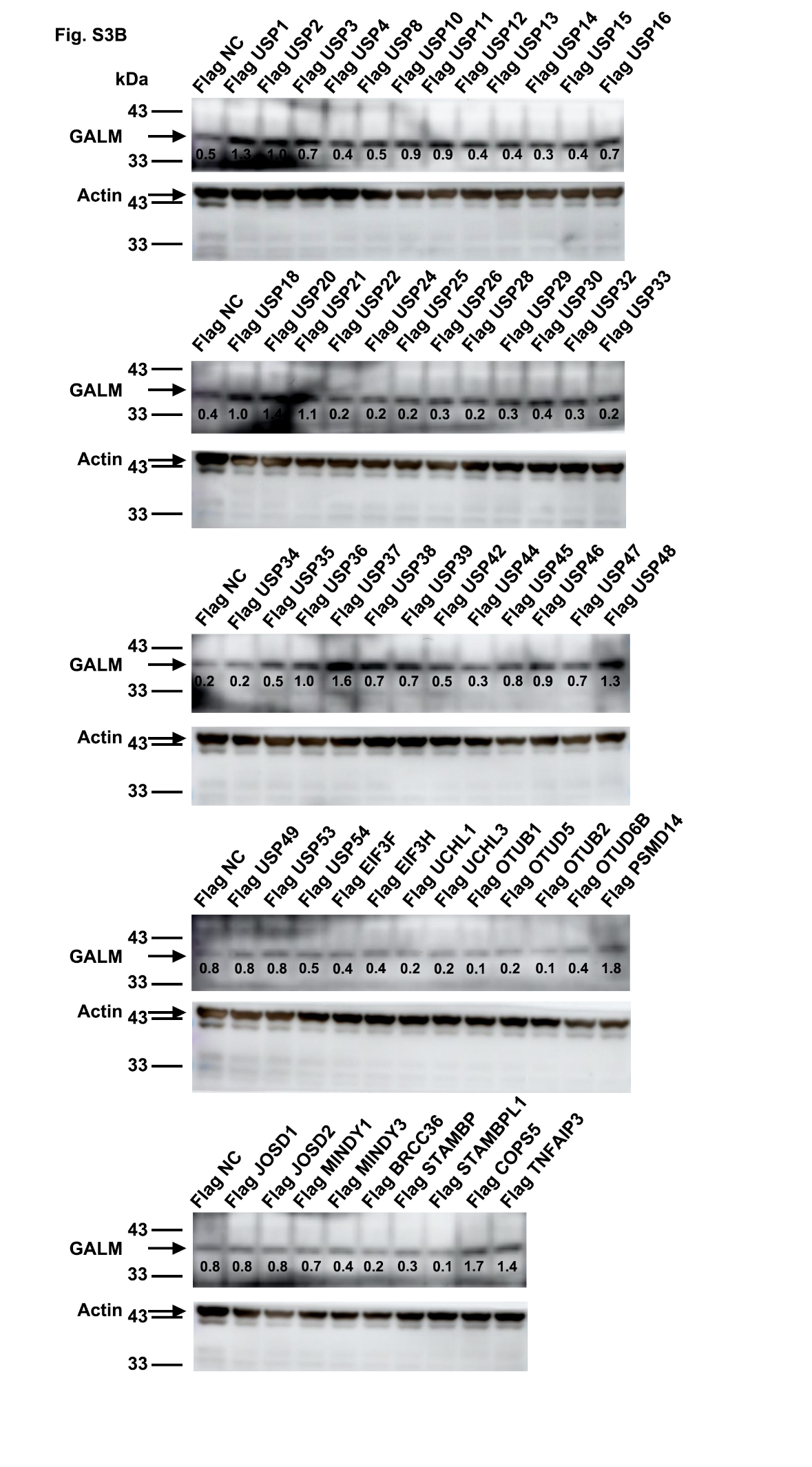


**Figure S5. Original Western blots with molecular weight markers.**

**Table S1. Detail information of 11 glucose metabolism-related pathways.**

| 11 glucose metabolism-related pathways |
| --- |
| \| GLYCOLYSIS_GLUCONEOGENESIS \| \| --- \| \| CITRATE_CYCLE_TCA_CYCLE \| \| PENTOSE_PHOSPHATE_PATHWAY \| \| PENTOSE_AND_GLUCURONATE_INTERCONVERSIONS \| \| FRUCTOSE_AND_MANNOSE_METABOLISM \| \| GALACTOSE_METABOLISM \| \| STARCH_AND_SUCROSE_METABOLISM \| \| N_GLYCAN_BIOSYNTHESIS \| \| OTHER_GLYCAN_DEGRADATION \| \| O_GLYCAN_BIOSYNTHESIS \| \| AMINO_SUGAR_AND_NUCLEOTIDE_SUGAR_METABOLISM \| |

**Table S2. Detail information of 289 GMGs.**

| Genes |
| --- |
| ACSS2, GCK, PGK2, PGK1, PDHB, PDHA1, PDHA2, PGM2, TPI1, ACSS1, FBP1, ADH1B, HK2, ADH1C, HK1, HK3, ADH4, PGAM2, ADH5, PGAM1, ADH1A, ALDOC, ALDH7A1, LDHAL6B, PKLR, LDHAL6A, ENO1, PKM, PFKP, BPGM, PCK2, PCK1, ALDH1B1, ALDH2, ALDH3A1, AKR1A1, FBP2, PFKM, PFKL, LDHC, GAPDH, ENO3, ENO2, PGAM4, ADH7, ADH6, LDHB, ALDH1A3, ALDH3B1, ALDH3B2, ALDH9A1, ALDH3A2, GALM, ALDOA, DLD, DLAT, ALDOB, G6PC2, LDHA, G6PC, PGM1, GPI, IDH3B, DLST, CS, SUCLG2P2, FH, SDHD, OGDH, SDHB, IDH3A, SDHC, IDH2, IDH1, ACO1, ACLY, MDH2, MDH1, OGDHL, PC, SDHA, SUCLG1, SUCLA2, SUCLG2, IDH3G, ACO2, RPE, RPIA, PGLS, PRPS2, TALDO1, TKT, TKTL2, PGD, RBKS, H6PD, RPEL1, PRPS1L1, PRPS1, DERA, G6PD, TKTL1, UGT1A10, UGT1A8, UGT1A7, UGT1A6, UGT2B28, UGT1A5, CRYL1, UGDH, UGT2A1, GUSB, UGT1A9, DCXR, DHDH, UGT2B11, UGP2, XYLB, UGT2B10, AKR1B1, UGT2B7, UGT2B4, UGT2A3, UGT1A4, UGT2B17, UGT1A1, UGT1A3, UGT2B15, MPI, PMM2, PMM1, GMDS, PFKFB4, MTMR6, PHPT1, PFKFB3, FCSK, PFKFB2, MTMR1, PFKFB1, AKR1B10, FPGT, KHK, MTMR2, MTMR7, GALK1, GLB1, GALE, B4GALT1, LALBA, MGAM, GALT, GLA, GANC, LCT, GALK2, B4GALT2, GAA, ENPP3, ENPP1, AMY2B, UXS1, GYS1, GYS2, GBA3, TREH, AMY1A, AMY1B, AMY1C, AMY2A, PYGB, PYGM, PYGL, ALG13, DOLPP1, RPN1, ALG14, MAN1B1, ALG3, MGAT5, RPN2, STT3A, MGAT3, DAD1, MGAT2, ALG12, TUSC3, MAN1C1, DPM2, DPM1, GANAB, ALG1, MGAT4A, ALG10B, STT3B, MAN1A2, ALG10, ALG11, ALG8, ALG2, ENGASE, MANBA, MAN2B1, GBA, NEU4, NEU2, NEU1, FUCA1, FUCA2, AGA, MAN2C1, MAN2B2, NEU3, HEXB, HEXA, GALNT4, GALNT15, GALNTL5, GALNT6, GALNT5, GALNT16, GALNTL6, GALNT13, GCNT3, GALNT10, ST6GALNAC1, GALNT9, GALNT7, GCNT4, GALNT11, GCNT1, GALNT8, GALNT14, B3GNT6, C1GALT1, GALNT18, GALNT12, GALNT2, GALNT3, GALNT17, GALNT1, ST3GAL2, ST3GAL1, GFPT2, AMDHD2, GNPDA2, GNPNAT1, NANS, CYB5R3, GFPT1, NANP, CYB5R1, GNE, PGM2L1, AGL, SI, GBE1, TSTA3, SORD, GMPPA, GMPPB, B4GALT5, C1GALT1C1, DPAGT1, RFT1, DPM3, DDOST, MGAT4B, ALG6, MAN2A2, MAN1A1, MAN2A1, ST6GAL1, B4GALT3, ALG5, MGAT5B, ALG9, MOGS, FUT8, MGAT1, NAGK, CHIT1, UAP1, RENBP, CHIA, NPL, CMAS, PGM3, GNPDA1 |

**Table S3. The detail correlation coefficients between DUBs and GALM.**

| Deubiquitinases (DUBs) | P value | R |
| --- | --- | --- |
| USP1 | 0 | 0.41 |
| USP2 | 0.76 | 0.012 |
| USP3 | 3.6$e^{-6}$ | -0.18 |
| USP4 | 0 | 0.44 |
| USP8 | 2.7$e^{-11}$ | 0.25 |
| USP10 | 0.0055 | -0.11 |
| USP11 | 2.4$e^{-46}$ | -0.51 |
| USP12 | 5.9$e^{-8}$ | -0.21 |
| USP13 | 1.4$e^{-5}$ | -0.17 |
| USP14 | 0 | 0.36 |
| USP15 | 0 | 0.48 |
| USP16 | 0.015 | 0.093 |
| USP18 | 0 | 0.37 |
| USP20 | 5$e^{-23}$ | -0.37 |
| USP21 | 0.2 | -0.049 |
| USP22 | 3.6$e^{-9}$ | -0.22 |
| USP24 | 0.81 | -0.009 |
| USP25 | 0.11 | 0.061 |
| USP26 | 0.68 | 0.016 |
| USP28 | 2.7$e^{-11}$ | 0.25 |
| USP29 | 1.1$e^{-7}$ | 0.2 |
| USP30 | 3.8$e^{-6}$ | -0.18 |
| USP32 | 0.08 | -0.067 |
| USP33 | 3.6$e^{-15}$ | 0.3 |
| USP34 | 1$e^{-20}$ | -0.35 |
| USP35 | 7.4$e^{-6}$ | -0.17 |
| USP36 | 0.04 | -0.079 |
| USP37 | 0.79 | 0.01 |
| USP38 | 0.00063 | 0.13 |
| USP39 | 1.1$e^{-12}$ | 0.27 |
| USP42 | 1.8$e^{-8}$ | 0.21 |
| USP44 | 0.00084 | -0.13 |
| USP45 | 0.00018 | 0.14 |
| USP46 | 7.7$e^{-15}$ | -0.29 |
| USP47 | 3.4$e^{-8}$ | -0.21 |
| USP48 | 0 | 0.32 |
| USP49 | 2.3$e^{-6}$ | -0.18 |
| USP53 | 0 | 0.35 |
| USP54 | 5.2$e^{-33}$ | -0.44 |
| EIF3F | 9.4$e^{-11}$ | -0.24 |
| EIF3H | 2$e^{-11}$ | -0.25 |
| UCHL1 | 0.3 | -0.04 |
| UCHL3 | 0 | 0.41 |
| OTUB1 | 1.4$e^{-18}$ | -0.33 |
| OTUB2 | 0.0019 | -0.12 |
| OTUD5 | 5.7$e^{-10}$ | -0.23 |
| OTUD6B | 0.039 | 0.079 |
| PSMD14 | 1.3$e^{-15}$ | 0.3 |
| JOSD1 | 0.15 | 0.056 |
| JOSD2 | 0 | 0.32 |
| MINDY1 | 1.5$e^{-15}$ | -0.3 |
| MINDY3 | 1.7$e^{-32}$ | -0.43 |
| BRCC36 | 6.1$e^{-13}$ | 0.27 |
| STAMBP | 1$e^{-6}$ | -0.19 |
| STAMBPL1 | 6.3$e^{-7}$ | -0.19 |
| COPS5 | 4.3$e^{-9}$ | 0.22 |
| TNFAIP3 | 0 | 0.52 |

**Table S4. The information of multiple data sets.**

| Data sets | Contents |
| --- | --- |
| Group A | USP18, TNFAIP3, EIF3H, USP39, USP29, USP38, OTUD6B, STAMBP |
| Group B | USP1, USP4, USP8, USP14, USP15, USP16, USP18, USP28, USP33, USP38, USP39, USP42, USP45, USP48, USP53, UCHL3, OTUD6B, PSMD14, JOSD2, BRCC36, COPS5, TNFAIP3 |
| Group C | USP1, USP2, USP3, USP10, USP11, USP18, USP20, USP21, USP36, USP37, USP38, USP39, USP46, USP48, PSMD14, COPS5, TNFAIP3 |
| Group A & Group B & Group C | USP18, TNFAIP3, USP39, USP38 |

**Table S5. Detail sequences of the siRNAs purchased.**

| GALM siRNAs | Sequences |
| --- | --- |
| si1GALM-255 | 5’-GCCAUACUUUGGAGCAGUUTT-3’,  5’-AACUGCUCCAAAGUAUGGCTT-3’ |
| si2GALM-341 | 5’-CCAUUAACAAGGAACCCAATT-3’,  5’-UUGGGUUCCUUGUUAAUGGTT-3’; |
| si3GALM-569 | 5’-CCAACCAUUCUUACUUCAATT-3’,  5’-UUGAAGUAAGAAUGGUUGGTT-3’ |

**Table S6. Sequences of target gene-specific primers.**

| Primers | Sequences |
| --- | --- |
| GALM-forward primer  GALM-reverse primer | 5’-TGGGTGACATACACCCTGGA-3’  5’-CTGGGGCAACTTCTCCTGTA-3’ |
| ACTIN-forward primer  ACTIN-reverse primer | 5’-CTCCATCCTGGCCTCGCTGT-3’  5’-GCTGTCACCTTCACCGTTCC-3’ |
