# Supplementary figures and images for: Comprehensive Analyses of Glucose Metabolism in Glioma Reveal the Glioma-Promoting Effect of GALM
Source: Front Cell Dev Biol. 2022 Jan 20;9:717182. doi: 10.3389/fcell.2021.717182 (PMC8811465; doi:10.3389/fcell.2021.717182)

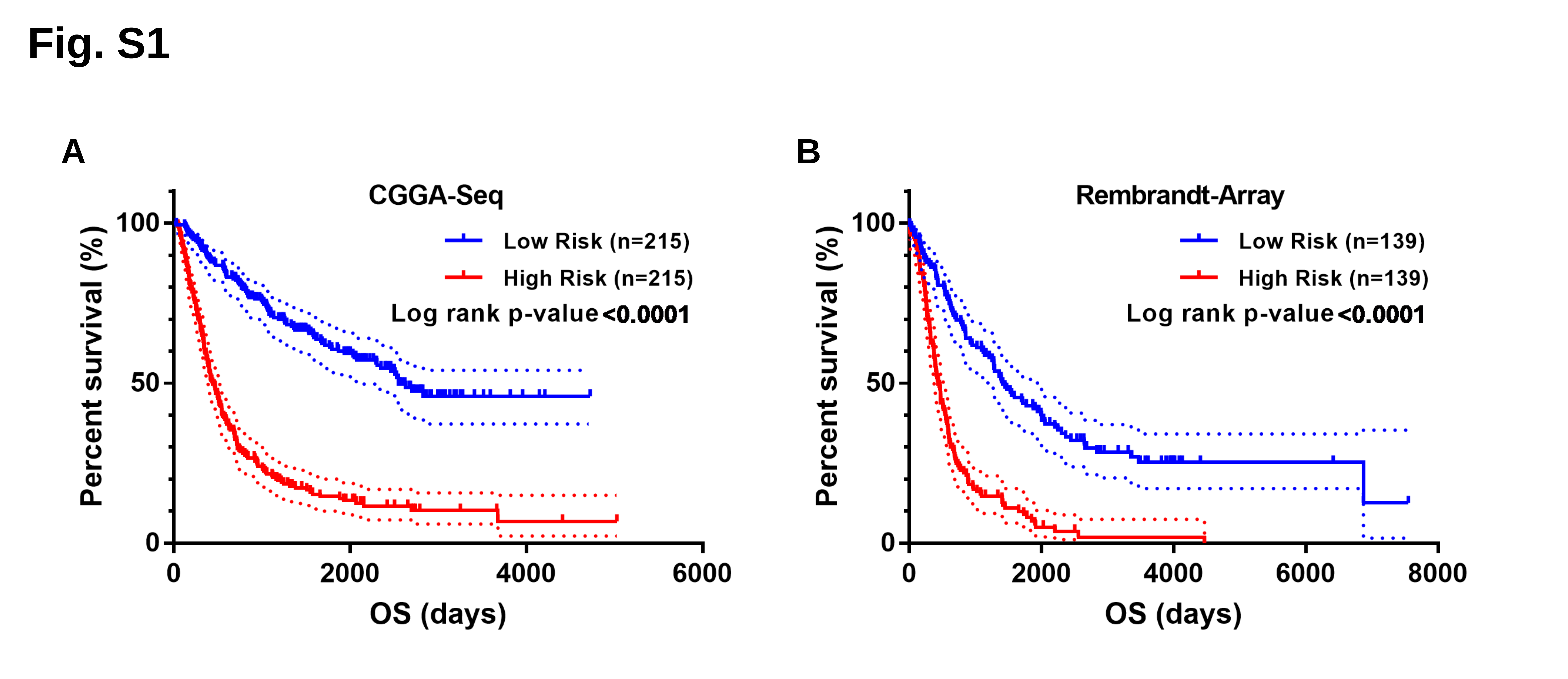

Supplement: Supplementary file 1 [file DataSheet1.ZIP › Supplementary Information/Fig. S1.tif]

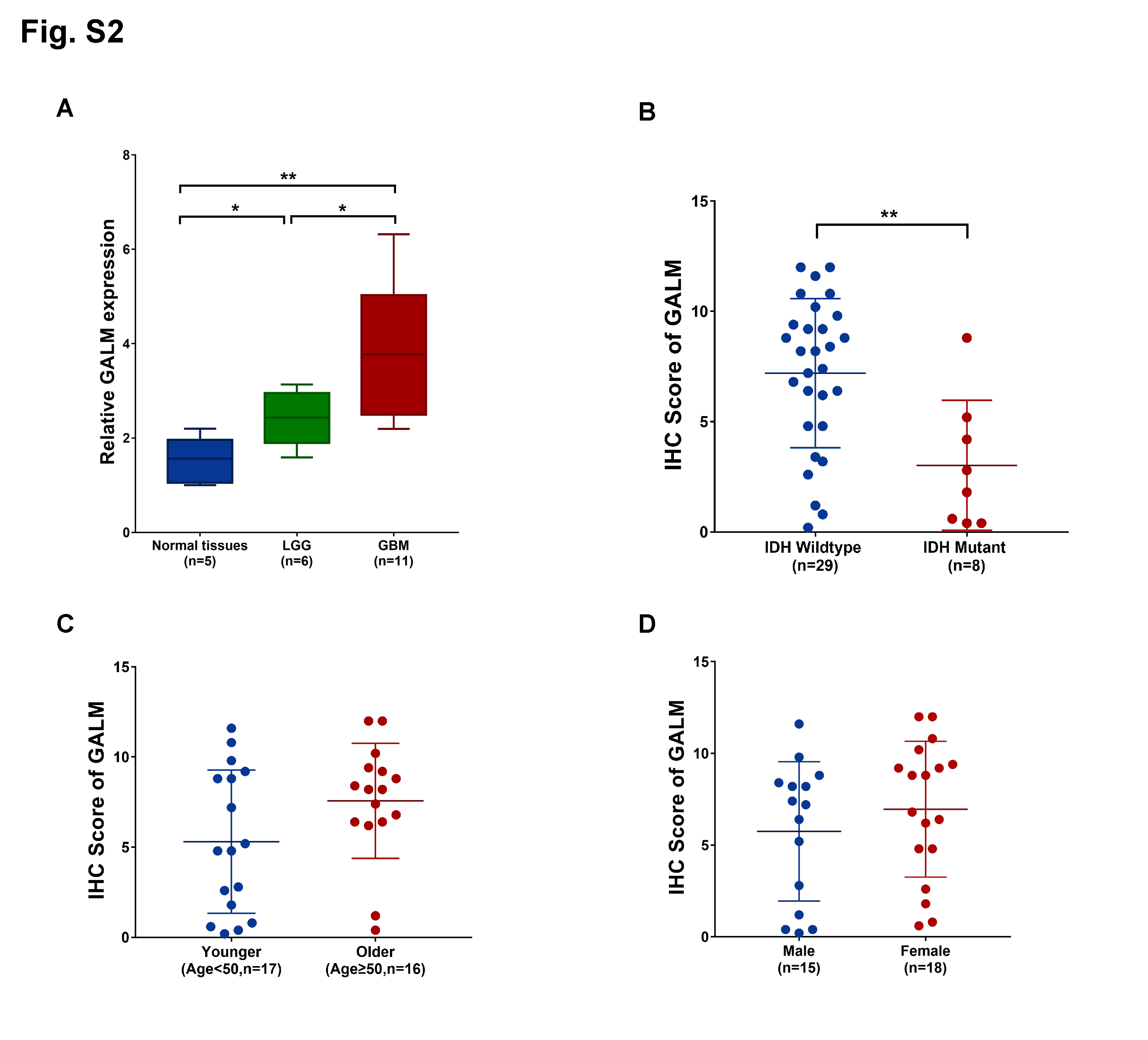

Supplement: Supplementary file 1 [file DataSheet1.ZIP › Supplementary Information/Fig. S2.tiff]

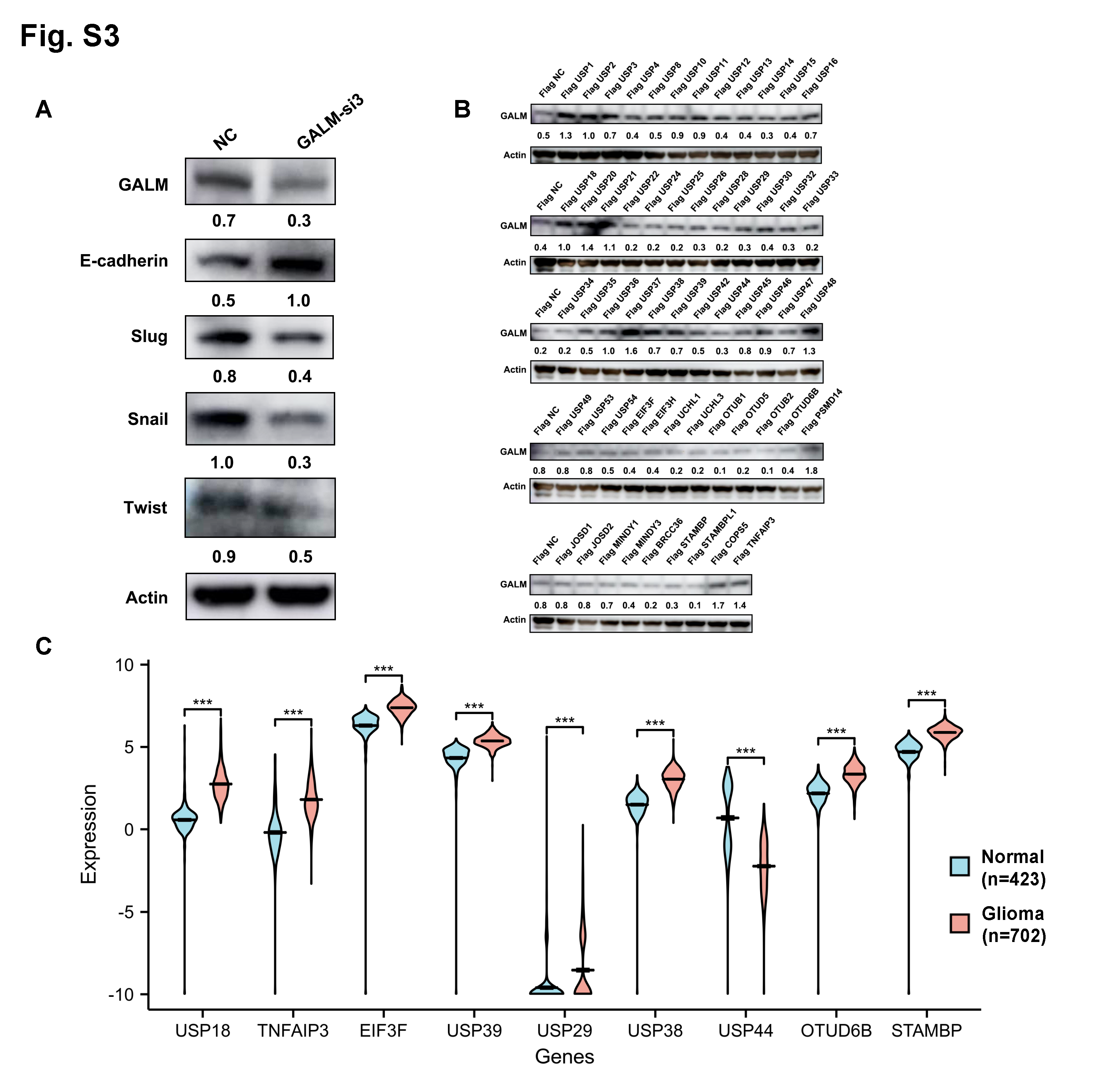

Supplement: Supplementary file 1 [file DataSheet1.ZIP › Supplementary Information/Fig. S3.tif]

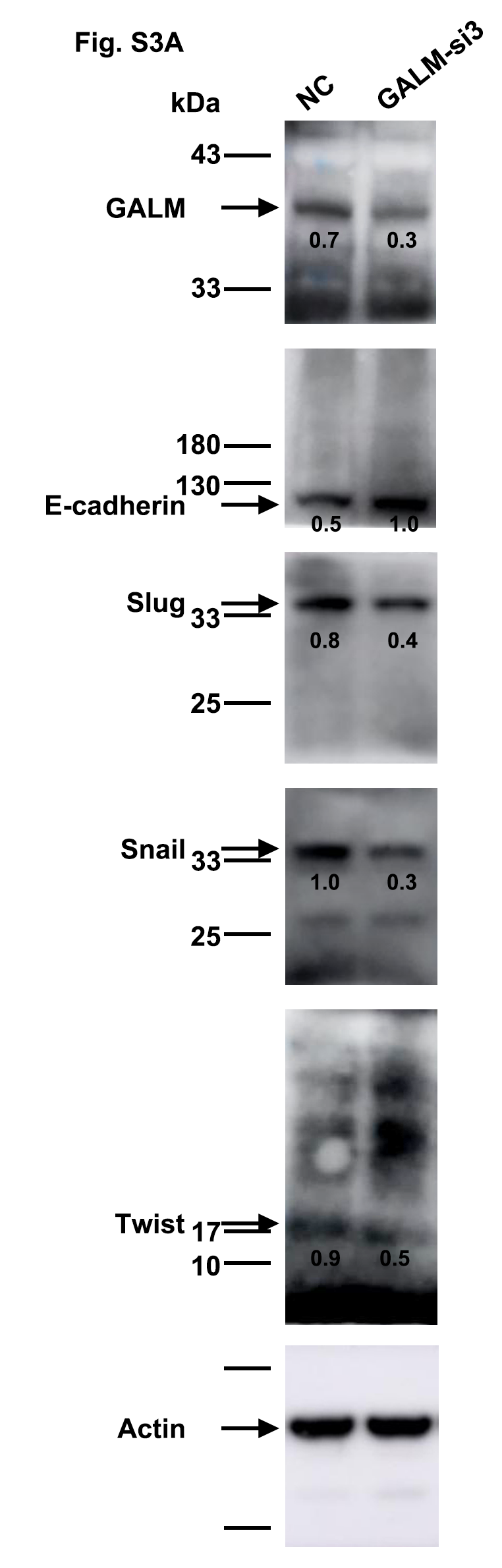

Supplement: Supplementary file 1 [file DataSheet1.ZIP › Supplementary Information/Fig. S3A.tif]

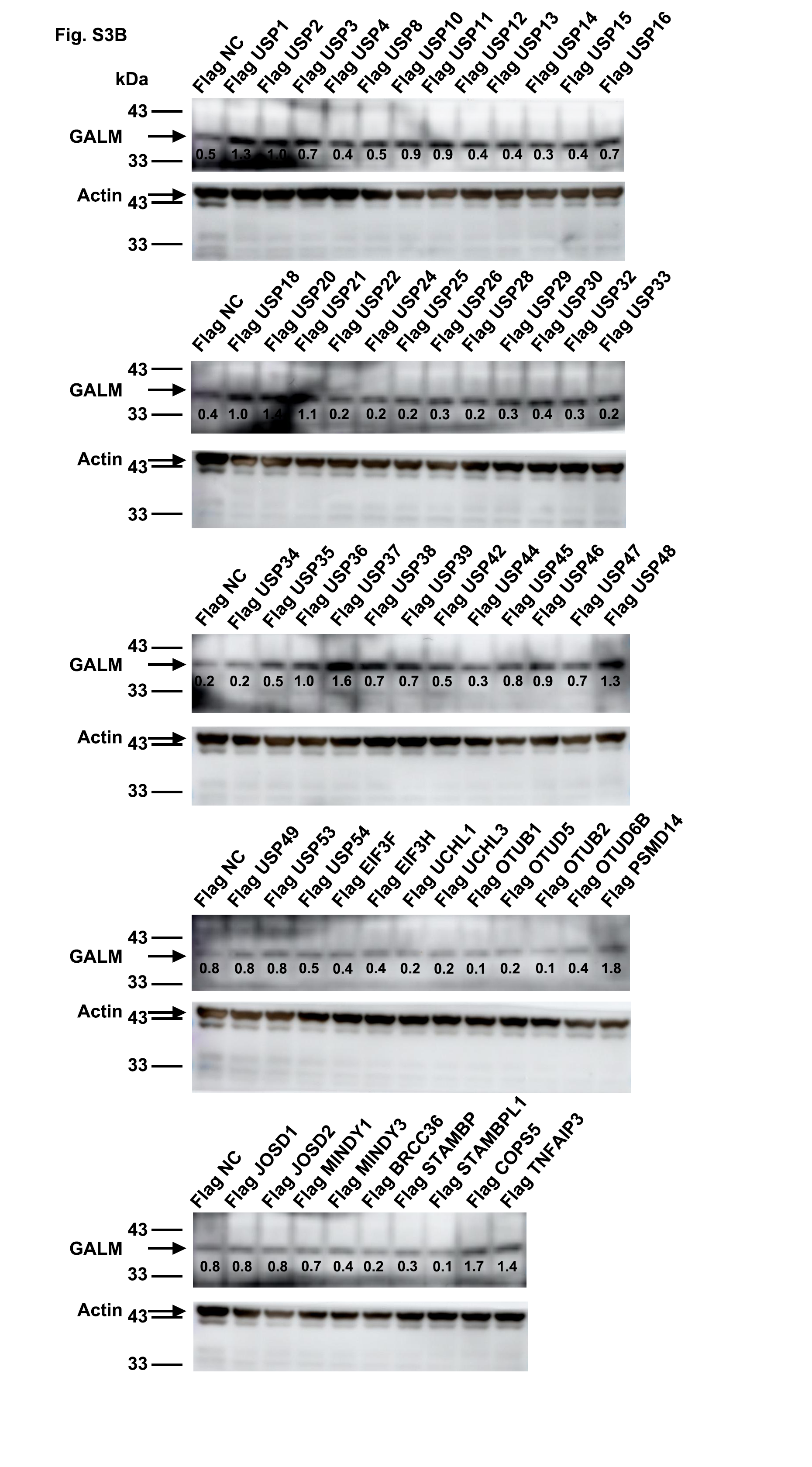

Supplement: Supplementary file 1 [file DataSheet1.ZIP › Supplementary Information/Fig. S3B.tif]

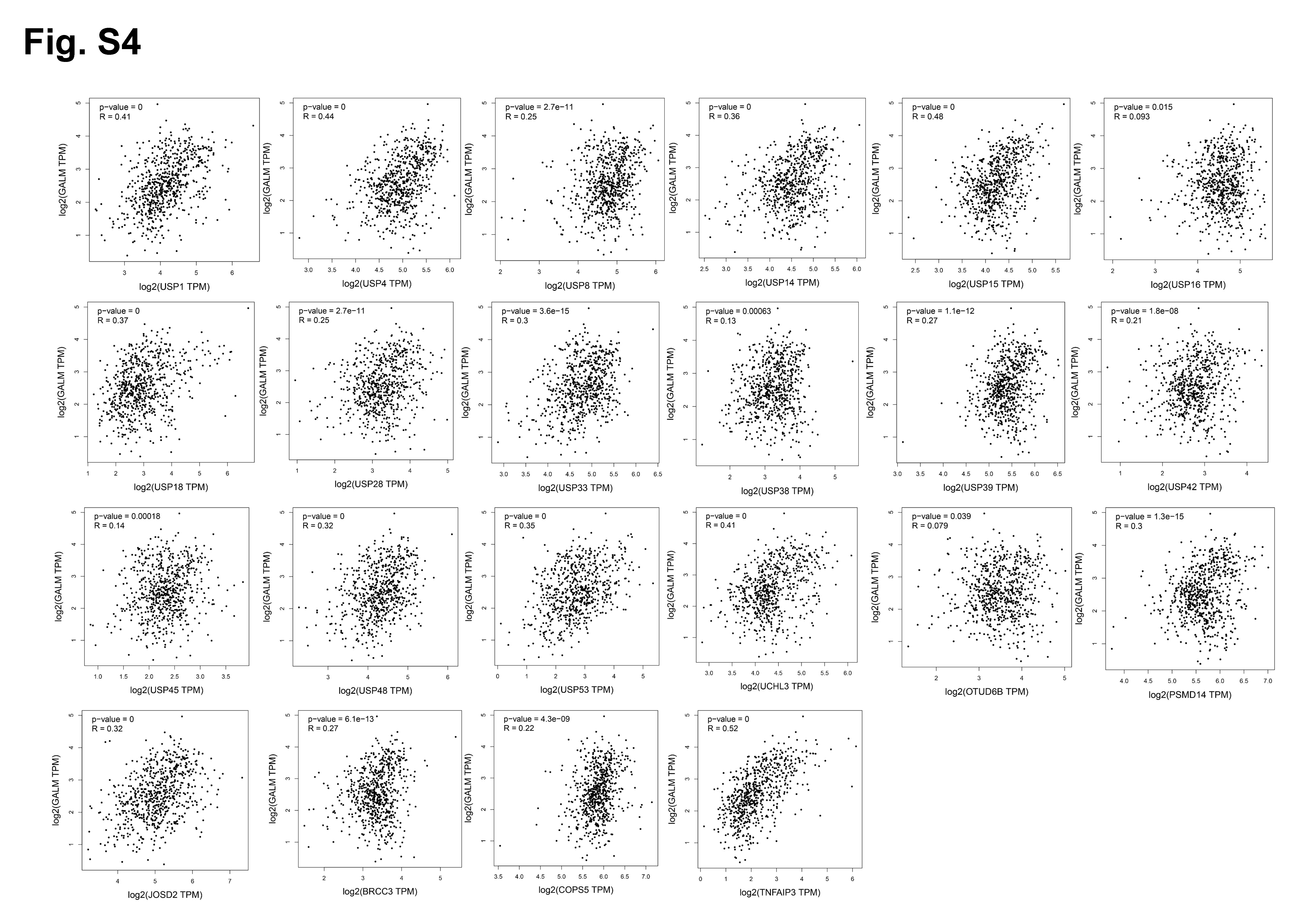

Supplement: Supplementary file 1 [file DataSheet1.ZIP › Supplementary Information/Fig. S4.tiff]

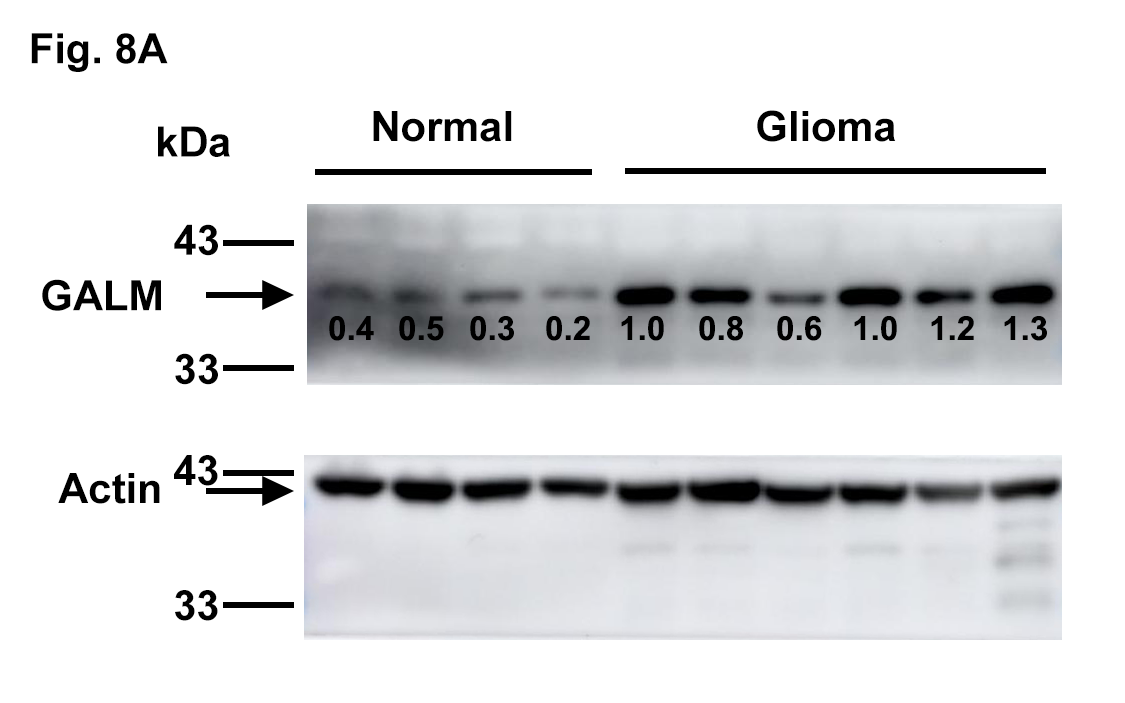

Supplement: Supplementary file 1 [file DataSheet1.ZIP › Supplementary Information/Figure. 8A.tif]

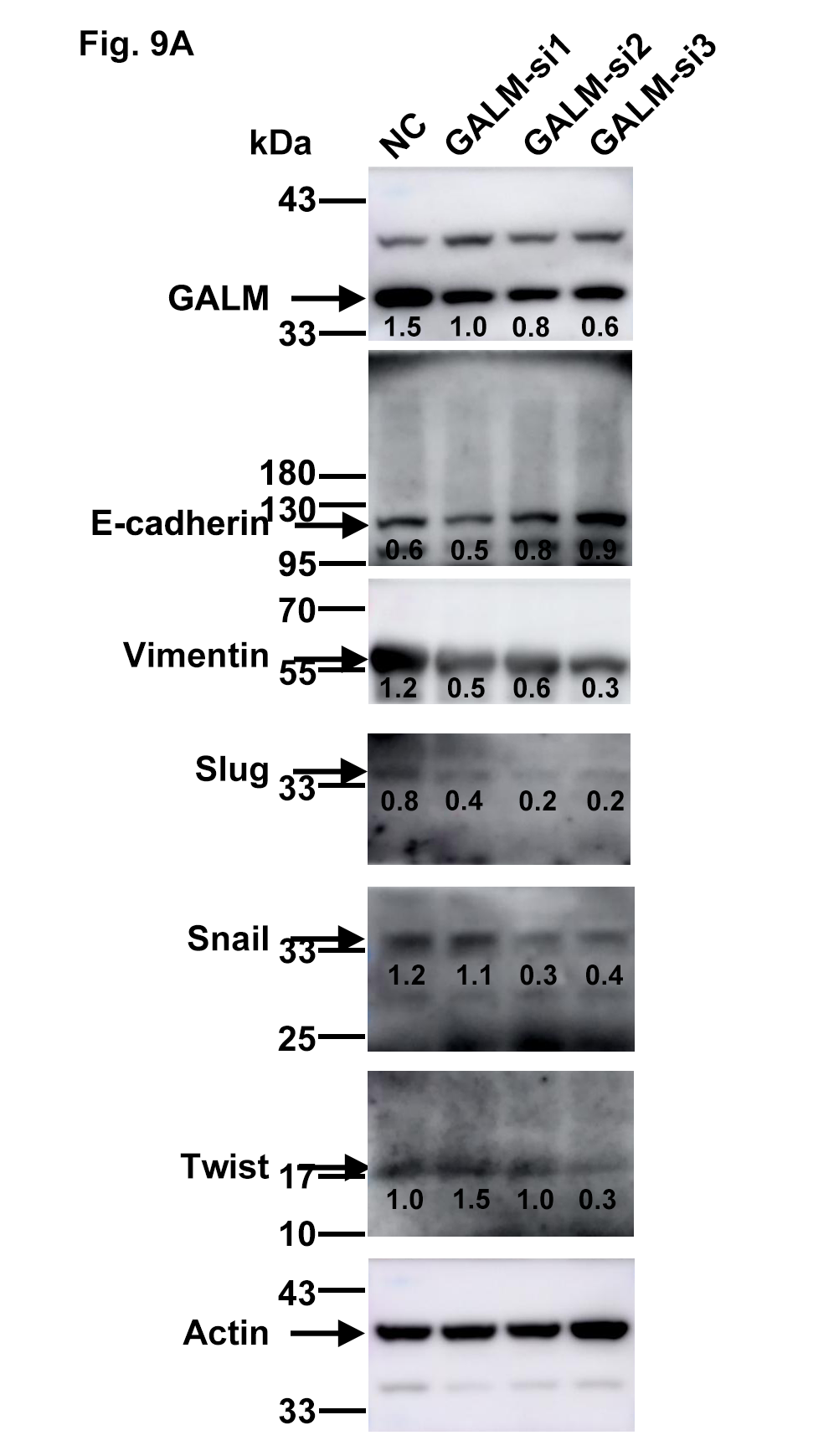

Supplement: Supplementary file 1 [file DataSheet1.ZIP › Supplementary Information/Figure. 9A.tif]

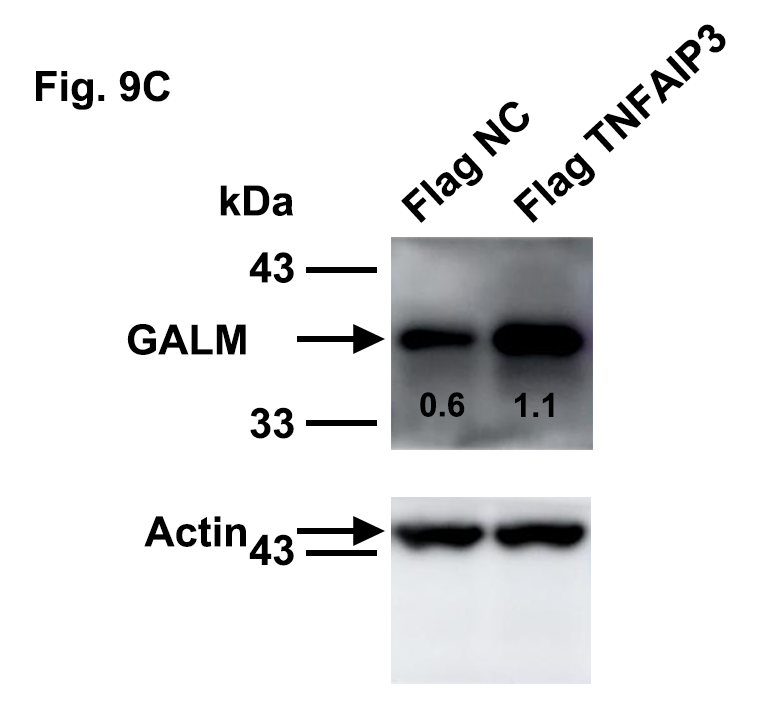

Supplement: Supplementary file 1 [file DataSheet1.ZIP › Supplementary Information/Figure. 9C.tif]
